# Oxygen‐Vacancy Abundant Ultrafine Co_3_O_4_/Graphene Composites for High‐Rate Supercapacitor Electrodes

**DOI:** 10.1002/advs.201700659

**Published:** 2018-01-15

**Authors:** Shuhua Yang, Yuanyue Liu, Yufeng Hao, Xiaopeng Yang, William A. Goddard, Xiao Li Zhang, Bingqiang Cao

**Affiliations:** ^1^ Materials Center for Energy and Photoelectrochemical Conversion School of Material Science and Engineering University of Jinan Jinan 250022 China; ^2^ Materials and Process Simulation Center California Institute of Technology Pasadena CA 91125 USA; ^3^ Department of Mechanical Engineering, and Texas Materials Institute The University of Texas at Austin Austin TX 78712 USA; ^4^ National Laboratory of Solid State Microstructures College of Engineering and Applied Sciences and Collaborative Innovation Center of Advanced Microstructures Nanjing University Nanjing 210093 China; ^5^ School of Materials Science and Engineering and State Centre for International Cooperation on Designer Low‐Carbon & Environmental Materials Zhengzhou University Zhengzhou 450001 China

**Keywords:** graphene, laser irradiation, oxygen vacancies, supercapacitors, ultrafine Co_3_O_4_ nanoparticles

## Abstract

The metal oxides/graphene composites are one of the most promising supercapacitors (SCs) electrode materials. However, rational synthesis of such electrode materials with controllable conductivity and electrochemical activity is the topical challenge for high‐performance SCs. Here, the Co_3_O_4_/graphene composite is taken as a typical example and develops a novel/universal one‐step laser irradiation method that overcomes all these challenges and obtains the oxygen‐vacancy abundant ultrafine Co_3_O_4_ nanoparticles/graphene (UCNG) composites with high SCs performance. First‐principles calculations show that the surface oxygen vacancies can facilitate the electrochemical charge transfer by creating midgap electronic states. The specific capacitance of the UCNG electrode reaches 978.1 F g^−1^ (135.8 mA h g^−1^) at the current densities of 1 A g^−1^ and retains a high capacitance retention of 916.5 F g^−1^ (127.3 mA h g^−1^) even at current density up to 10 A g^−1^, showing remarkable rate capability (more than 93.7% capacitance retention). Additionally, 99.3% of the initial capacitance is maintained after consecutive 20 000 cycles, demonstrating enhanced cycling stability. Moreover, this proposed laser‐assisted growth strategy is demonstrated to be universal for other metal oxide/graphene composites with tuned electrical conductivity and electrochemical activity.

## 1 Introduction

Supercapacitors (SCs) are becoming a critical member of energy storage systems and have been widely used in consumer electronics, hybrid electric vehicles, uninterruptable power supplies, and so on.^1^, ^2^, ^3^ SCs can be classified into two categories: electrical double‐layer capacitors (EDLCs) where charge is stored by charge separation at the electrode–electrolyte interface, and pseudocapacitors that store charge by fast Faradic reactions. EDLCs employing carbon materials have low capacitance, whereas pseudocapacitors utilizing metal oxides (NiO, Co_3_O_4_, MnO_2_, and RuO_2_) and conducting polymers exhibit low rate capability and short cycle life.^4^, ^5^, ^6^, ^7^, ^8^, ^9^, ^10^ A strategy to overcome these problems is to grow or anchor metal oxides or conducting polymers onto carbon‐based material to obtain novel composite electrode materials, which derive their benefits from synergistic effects. Despite the promising results and significant progress in this field, rational synthesis of such electrode materials with controllable conductivity and electrochemical activity is still the topical challenge for high‐performance SCs.

The Co_3_O_4_/graphene composites have become a highly promising electrode material for high‐performance SCs,^11^, ^12^ in which Co_3_O_4_ has a high theoretical‐specific capacitance (3560 F g^−1^), low cost, and great reversibility, while the graphene sheets offer excellent electron conductive routes, ultralarge‐specific surface area, and superior intrinsic mechanical strength. Many approaches are available for fabricating the Co_3_O_4_/graphene composites for the charge‐storage performance. Dong et al. and Liao et al. reported that the Co_3_O_4_/graphene hybrids were in situ synthesized by a simple hydrothermal procedure, which delivers a high‐specific capacitance and exhibits an excellent cycling stability.^12^, ^13^ Zou et al. synthesized mesoporous Co_3_O_4_ nanosheets on graphene foam through the electrodeposition and annealing process, in which the 3D hierarchical structure and the synergetic effect of Co_3_O_4_ and graphene enhance the rate of ion and electron transport in electrodes.^14^ Chen and Wang prepared a Co_3_O_4_/graphene nanostructures by the microwave irradiation method, which is used as the electrode for lithium ion batteries and shows an excellent rate capability and highly reversible large capacity.^15^ Unfortunately, there are only a few reports about the preparation of ultrafine Co_3_O_4_ nanoparticles on graphene,^16^, ^17^ which are considered as one of the most optimal structures for high‐performance SCs due to a high utilization rate of pseudocapacitive materials (Co_3_O_4_) and a short transport length of electrolyte ions and electrons in the composites.^18^, ^19^ Also, the in situ synthesis of such composite nanostructure with ultrafine Co_3_O_4_ nanoparticles, high conductive graphene, and tight coupling between those two remains challenging, and there are still somewhat limited (e.g., complicated process, pollution of organic additives). What's more, controlling the intrinsic conductivity of Co_3_O_4_ nanoparticles and their electrochemical activity is another great challenge in promoting the SCs performance.

In recent years, intentional creation of oxygen vacancies (*V*_O_) has been explored to improve the intrinsic conductivity and electrochemical activity of transition metal oxides, and thus to increase their SCs performance,^20^, ^21^, ^22^ where oxygen vacancies (*V*_O_) can change the geometric and electronic structures as well as the chemical properties of transition metal oxides. However, owing to the limitations of synthetic methods (e.g., chemical reduction, gas reduction, etc.),^19^, ^20^, ^23^, ^24^, ^25^, ^26^, ^27^, ^28^, ^29^ metal oxides with abundant oxygen vacancies, especially for ultrafine metal oxides nanoparticles/graphene composites, have been rarely fabricated. Thus, an innovative protocol is necessary for developing a unique ultrafine metal oxides nanoparticles/graphene nanostructure, in which appropriate oxygen vacancies in metal oxide were created.

Herein, we proposed a novel one‐step laser irradiation route through simultaneous laser‐induced reduction and fragmentation to prepare oxygen‐vacancy abundant and ultrafine Co_3_O_4_ nanoparticles/graphene (UCNG) composites with high rate capability (93.7%) for the first time. Without introducing any reducing agents and organic additives, ultrafine Co_3_O_4_ nanoparticles (≈10 nm) anchoring on graphene were obtained. Under laser irradiation, the graphene oxide (GO) was reduced to high conductive graphene through photothermal effects, accompanied the fragmentation (size reduction) of the porous Co_3_O_4_ nanorods (P‐Co_3_O_4_). More importantly, abundant oxygen vacancies were created on the ultrafine Co_3_O_4_ nanoparticles surface and the Co_3_O_4_ nanoparticles tightly anchored on the graphene, which significantly modifies the electric conductivity and the electrochemical activity of Co_3_O_4_ nanoparticles and hence enables the UCNG electrode with fast and reversible Faradaic reactions. The ultrafine Co_3_O_4_ nanoparticles/graphene composites are the promising candidates as the supercapacitor electrode. The capacitance of the composites reached 978.1 F g^−1^ (135.8 mA h g^−1^) at the current densities of 1 A g^−1^. It shows high rate capability, with more than 93.7% capacitance retention at even 10 A g^−1^. The superior performance also benefits from the surface midgap electronic states, which are enhanced by oxygen vacancies (*V*_O_) and facilitate the electrochemical charge transfer, as shown by our first‐principles calculations. The discovery in our paper not only establish a new route to prepare the Co_3_O_4_/graphene composites and shed light on their formation mechanism, but also opens a new avenue for realizing high‐performance SCs electrodes, especially for rate capability.

## 2 Results and Discussion

### 2.1 Proposed Growth Strategy

**Figure 1:**
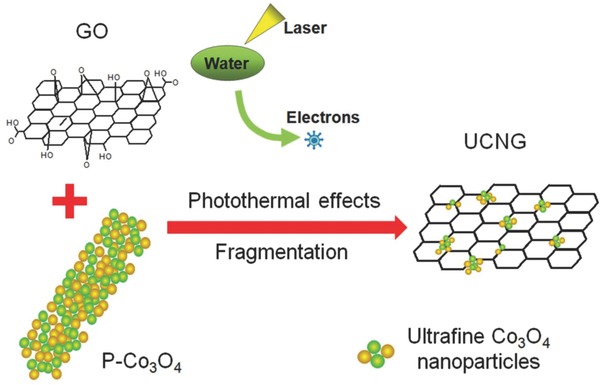
Schematic diagram of the formation mechanism for the UCNG composites. Under laser irradiation, the GO was reduced to LG through photothermal effects, while the porous Co_3_O_4_ nanorods completely fragmented into ultrafine Co_3_O_4_ nanoparticles and then were tightly anchored on LG surface.

The proposed laser‐assisted growth strategy for the UCNG composites is illustrated in **Figure** **1**. The most special feature of the laser irradiation is the extreme nonequilibrium process produced by the transient laser excitation of the mixture in liquid medium.^30^, ^31^, ^32^ Usually, the laser–target interaction in liquid medium would lead to the resizing/reshaping of the suspended particles and/or heat‐induced reaction.^33^, ^34^, ^35^ We chose as‐prepared porous Co_3_O_4_ nanorods (P‐Co_3_O_4_) and GO as the mixed precursor, and ultrapure water as solvent. During krypton difluoride (KrF) excimer laser (248 nm, 5 ns, 10 Hz) irradiation process, the fragmentation (size reduction) of the P‐Co_3_O_4_ nanorods occurs in the liquid, which may be driven by the laser–nanorods interaction‐induced plasma^34^, ^36^, ^37^ and/or photothermal effect (Figure S1, Supporting Information) as Co_3_O_4_ can also absorb this laser. Meanwhile, GO was transformed into reduced graphene oxide (called laser‐reduced graphene (LG) for clarity) after the laser irradiation, mainly because its large surface area with strong optical absorption can result in a rapid temperature rise in GO (photothermal effects) and subsequently lead to a significant temperature rise of water, which has been demonstrated to be a facile reduction method for the synthesis of graphene by recent reports.^8^, ^35^, ^38^, ^39^ Electrons can be generated from water in such extreme nonequilibrium reaction system under the laser irradiation,^40^, ^41^ which may also play a role in reducing GO to LG. Moreover, the LG containing multiple defect sites can provide efficient nucleation sites for anchoring the ultrafine Co_3_O_4_ nanoparticles. As a result, the coupling of the fragmentation of the P‐Co_3_O_4_ with the reduction of GO to LG through laser irradiation could provide a novel method for the synthesis of the UCNG composites. For the yield and production rate of the laser processing, it has been demonstrated that the laser fluence dominates nanomaterials throughput for many materials.^42^ Therefore, the yield of nanomaterials through laser synthesis is increasing with the steadily increasing output power of pulsed laser systems and significantly reducing the investment cost per laser power. In addition, the pulsed laser systems is another contributing factor for upscaling purposes.^42^

### 2.2 Morphology and Structural Characterization

**Figure 2:**
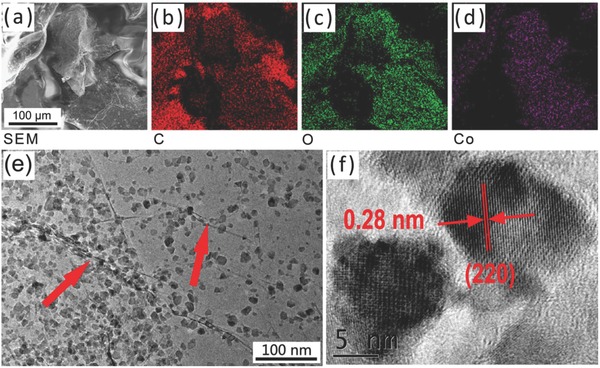
a–d) SEM image of UCNG composites and the corresponding elemental mapping of carbon, oxygen, and cobalt. e) TEM image and f) HRTEM image of UCNG composites.

A number of Co_3_O_4_ nanoparticles/graphene composites prepared under various laser parameters were surveyed to prove the above strategy and optimize the preparation condition associated with excellent electrochemical properties for SCs electrodes applications (Figures S2 and S3, Tables S1 and S2, Supporting Information). The optimized product (UCNG composites) with ultrafine Co_3_O_4_ nanoparticles and high conductive graphene was selected and discussed below. The morphology and microstructure of the UCNG were examined by the scanning electron microscopy (SEM) and transmission electron microscopy (TEM). The SEM images (**Figure** **2**a–d and Figure S4a,b, Supporting Information) show that the ultrafine Co_3_O_4_ nanoparticles are homogeneously distributed in graphene sheets. In the UCNG composites, the ultrafine Co_3_O_4_ nanoparticles prevent the restacking of graphene sheets for higher surface areas and maintain the intrinsic conductivity, whereas the graphene sheets avoid the agglomeration of the ultrafine Co_3_O_4_ nanoparticles due to the anchoring effect. The TEM image further indicates that the 2D graphene sheets are well decorated by numerous ultrafine Co_3_O_4_ nanoparticles with sizes of ≈10 nm and a slight fusion (Figure 2e, Figures S4c,d, S5, and S6, Supporting Information). Also, the graphene can be clearly observed as indicated by the arrow in Figure 2e. Such ultrafine Co_3_O_4_ nanoparticles are attributed to laser fragmentation mechanism in transforming the P‐Co_3_O_4_ into the small size Co_3_O_4_ particles,^34^ which was certified by the TEM images of Co_3_O_4_ particles before and after laser irradiation (Figure S7, Supporting Information and Figure 2e). Laser‐induced photoelectron ejection leaves positive charges on the surface of the P‐Co_3_O_4_,^37^ which drives electrostatic repulsion among different parts in the nanorods and then induces the fragmentation and the formation of smaller particles. The neck formation (slight fusion) between adjacent Co_3_O_4_ particles may be mainly caused by laser melting.^43^ The tight connection between graphene sheets and Co_3_O_4_ particles is ascribed to the coupling of the photothermal effects during the laser irradiation of the mixture solution, which assists in anchoring the ultrafine Co_3_O_4_ nanoparticles to the defect sites created by the laser reduction of GO.^38^ The high‐resolution TEM (HRTEM) image (Figure 2f) shows the crystal lattice fringes with an interlayer distance of 0.28 nm, which is in accordance with the (220) plane of Co_3_O_4_. The few‐layer graphene combined with ultrafine Co_3_O_4_ nanoparticles should be expected to exhibit higher effective surface area and shorter ion diffusion length, benefiting the high‐performance supercapacitor.^18^, ^19^ Even after long time sonication, there is no obvious exfoliation of the Co_3_O_4_ nanoparticles from the graphene, confirming strong anchoring between Co_3_O_4_ nanoparticles and graphene sheets, which not only preserve the high conductivity of the bulk electrode materials, but also significantly enhance the electrochemical activity of ultrafine Co_3_O_4_ nanoparticles during the charge/discharge process. The interaction between graphene and Co_3_O_4_ nanoparticles is further corroborated by X‐ray photoemission spectroscopy (XPS) and Fourier transform infrared (FTIR) spectroscopy (Figures S8, S9, Supporting Information).

**Figure 3:**
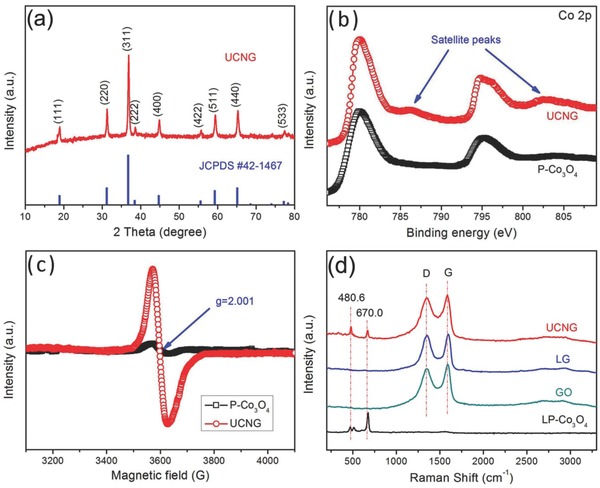
a) XRD patterns of UCNG composites. b) XPS survey spectra of UCNG composites and P‐Co_3_O_4_. c) Room‐temperature EPR spectra of UCNG composites and P‐Co_3_O_4_. d) Raman spectra of UCNG composites, LG, GO, and LP‐Co_3_O_4_.

The crystal structure of the UCNG composites, P‐Co_3_O_4_ and LP‐Co_3_O_4_, was characterized by X‐ray diffraction (XRD) in a scan range of 10°–80° (**Figure** **3**a and Figure S10, Supporting Information). The typical XRD diffractogram of the UCNG composites shows that all peaks can be indexed as the crystalline Co_3_O_4_ according to the JCPDS card No. 42‐1467. The diffraction peaks of carbon species were not detectable, indicating highly disordered and few‐layer graphene sheets in the UCNG composites because the ultrafine Co_3_O_4_ nanoparticles between the graphene sheets avoid the restacking and agglomeration of the graphene sheets.^44^ These results also agree with the electron microscopy investigation. Additionally, the P‐Co_3_O_4_ and LP‐Co_3_O_4_ were prepared as a control group. As can be seen in Figure S10 (Supporting Information), their diffraction lines are in line with the UCNG composites, indicating that the laser irradiation and the graphene introduction have no effect on the Co_3_O_4_ crystal structure.

To further investigate the oxidation status of cobalt, XPS measurements were carried out to examine both the P‐Co_3_O_4_ and the UCNG composites (Figure 3b). In the high‐resolution Co 2p spectrum, the peaks observed at 780.0 and 795.5 eV for the P‐Co_3_O_4_ (780.1 and 794.9 eV for the UCNG composites) can be assigned to the Co 2p_3/2_ and Co 2p_1/2_ spin‐orbital peaks of Co_3_O_4_, respectively.^12^, ^45^ Compared to the P‐Co_3_O_4_, the Co 2p peaks of the UCNG composites show two more obvious satellite peaks centered at about 786.3 and 802.5 eV, which are attributed to the Co^2+^ oxidation state, indicating that a part of the Co^3+^ ions was transformed to Co^2+^ and formed oxygen vacancies. A similar phenomenon of the transformation from higher valence metal ions to lower valence metal ions under laser irradiation is also found in our previous work,^40^ mainly attributing to the reducing action by using electrons generated in reaction system. According to the XRD and XPS results, we can infer that the surface of the Co_3_O_4_ in the UCNG composites is reduced by laser irradiation and plentiful oxygen vacancies on the surface are produced, while the main body of the nanoparticles is still the Co_3_O_4_ crystal. The peaks in the XPS spectrum of LP‐Co_3_O_4_ are similar to those of the UCNG composites (Figure S11, Supporting Information), indicating the oxygen vacancies are induced through laser irradiation and are not affected by the introduction of GO.

The electron paramagnetic resonance (EPR) was furthermore employed to investigate the oxygen vacancies in the Co_3_O_4_ among the UCNG composites (Figure 3c), which was proved to be an effective tool to examine unpaired electrons in materials.^46^, ^47^, ^48^ The UCNG composites display a symmetrical EPR signal at *g* = 2.001, while the EPR signal for the P‐Co_3_O_4_ is negligible. In addition, the LP‐Co_3_O_4_ shows a similar EPR spectrum to the UCNG composites (Figure S12, Supporting Information). The prominent resonance line of paramagnetic phase in the EPR absorption spectrum for the UCNG composites demonstrates that there are more Co^2+^ ions in the Co_3_O_4_ crystal, which further manifest the presence of oxygen vacancies.^49^ Although a large number of oxygen vacancies were formed in the ultrafine Co_3_O_4_ nanoparticles, the Co_3_O_4_ phase has not been altered as indicated by XRD (Figure 3a). Taken together, we can conclude that the oxygen vacancies were produced only on the surface of the Co_3_O_4_ in the UCNG composites. Oxygen vacancies have been proven to be a unique and efficient means for enhancing the electrical conductivity and electrochemical performance in metal oxides.^19^, ^20^, ^22^, ^24^, ^50^, ^51^ Thus, the UCNG composites are expected to improve the rate performance and specific capacitance of the electrode material in supercapacitors.

The local structures of the UCNG composites, LP‐Co_3_O_4_, GO, and LG were analyzed by Raman spectroscopy. As observed in Figure 3d, the characteristic D and G bands for GO, LG, and UCNG composites are obviously detected. The G peak represents the *E*_2g_ vibrational mode within aromatic carbon rings and the degree of graphitization for the area of excitation, while the D peak is ascribed to the small and isolated domains of aromaticity and the disorder of graphene. The weak 2D band (≈2700 cm^−1^) confirms the few‐layer graphene structures obtained by laser irradiation.^19^ In the Raman spectrum of LP‐Co_3_O_4_, three characteristic peaks at 470.0, 512.1, and 676.8 cm^−1^ were showed, which corresponds to the *E*_g_, *F* ^1^_2g_, and *A* ^1^_g_ modes of the crystalline Co_3_O_4_. It should be noted that the peaks at 480.6 and 603 cm^−1^ from Co_3_O_4_ can be observed in the UCNG composites. Compared to LP‐Co_3_O_4_, the obvious peak shift and disappearance for the Co_3_O_4_ particles in the UCNG composites could be attributed to the effective bonding between Co_3_O_4_ and graphene,^52^ further confirming the anchoring of the ultrafine Co_3_O_4_ nanoparticles on the graphene sheets. In addition, after the laser irradiation, the D/G ratio associated with both LG and UCNG composites is increased (from 0.93 for GO to 0.97 for LG and 0.95 for UCNG composites), indicating a high concentration of structure defects in the graphene lattice induced by laser irradiation.^53^ The increase of D/G ratio also indicates the reduction of GO under the laser irradiation, as the reduction can increase the number of small domains of aromaticity responsible for the D peak. A similar phenomenon in reduced graphene oxide samples was observed in previous reports.^54^, ^55^ The GO was reduced by laser, which is further corroborated by a color change before and after laser irradiation (Figure S13, Supporting Information). The defect sites on the LG nanosheets act as favorable nucleation sites for the ultrafine Co_3_O_4_ nanoparticles, which restricts their mobility and prevents the Ostwald ripening process, i.e., the formation of large Co_3_O_4_ particles and the aggregation of the nanoparticles during the synthesis processes. These results are consistent with the SEM and SEM results. In order to discover the effect of GO on the formation of the ultrafine Co_3_O_4_ nanoparticles, we conducted the control experiments with the same experimental conditions except in the absence of GO. It can be seen that the porous Co_3_O_4_ nanorods completely fragmented after laser irradiation, but most of ultrafine Co_3_O_4_ nanoparticles agglomerate and even fused into larger spherical particles (Figure S14, Supporting Information), which confirms that GO is necessary for the formation of the ultrafine Co_3_O_4_ nanoparticles.

### 2.3 Electrochemical Characterization

**Figure 4:**
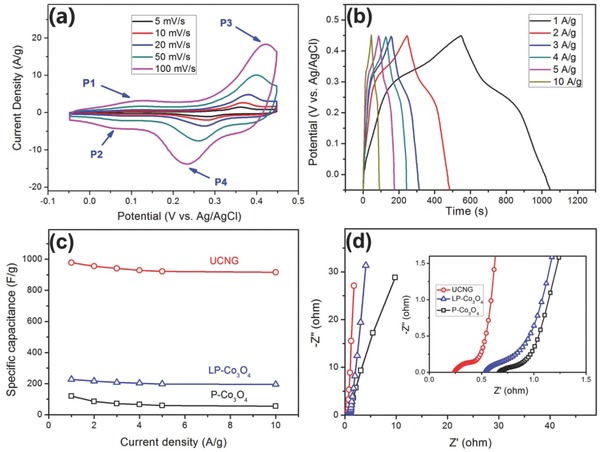
a) CV curves of the UCNG composites at different scan rates. b) GCD curves of the UCNG composites at various current densities. c) Specific capacitance of the UCNG composites, P‐Co_3_O_4_, and LP‐Co_3_O_4_ electrodes calculated from GCD curves as a function of current densities. d) EIS of the UCNG composites, P‐Co_3_O_4_, and LP‐Co_3_O_4_ electrodes.

We carried out cyclic voltammetry (CV), galvanostatic charge/discharge (GCD), and electrochemical impedance spectroscopy (EIS) measurements in a three‐electrode electrochemical cell with a platinum foil (1 cm × 1 cm) counterelectrode and an Ag/AgCl reference electrode in 2 m KOH aqueous solution. **Figure** **4**a presents typical CV curves of the UCNG composites at scan rates from 5 to 100 mV s^−1^ in a potential range of −0.05–0.45 V. Two obvious redox peaks are observed in every curve, indicating that the capacitances are mainly from pseudocapacitance characteristics due to Faradaic redox reactions. The anodic and cathodic peaks exhibit a symmetrical shape, demonstrating the high redox reversibility of the UCNG composites.^19^, ^56^ It has been demonstrated that the two couples of redox peaks are derived from the reversible transitions between Co_3_O_4_ and CoOOH (P1/P2) and between CoOOH and CoO_2_ (P3/P4). The surface redox reactions are presented as follows (Equations (1) and (2))^57^, ^58^

(1) $${\mathrm{Co}}_{3}O_{4}+{\mathrm{OH}}^{-}+H_{2}O↔3\mathrm{CoOOH}+e^{-}$$

(2) $$\mathrm{CoOOH}+{\mathrm{OH}}^{-}↔{\mathrm{CoO}}_{2}+H_{2}O+e^{-}$$

Figure 4b exhibits the GCD curves of the UCNG composites at different current densities. The profiles show symmetric charge/discharge curves with well‐defined plateaus, indicating a typical pseudocapacitance behavior and a high charge–discharge coulombic efficiency. The specific capacitance of the UCNG composites at different current densities is calculated from the discharge curves, which is well accepted that the GCD test is a more reasonable method than CV test.^59^ The specific capacitances of the UCNG composites at the current densities of 1, 2, 3, 4, 5, and 10 A g^−1^ are 978.1, 955.6, 941.3, 929.2, 921.8, and 916.5 F g^−1^ (135.8, 132.7, 130.7, 129.1, 128.0, and 127.3 mA h g^−1^), respectively. Figure 4c shows the variations of the specific capacitance for different electrode materials with various current densities from 1 to 10 A g^−1^. It was observed that the specific capacitance of the UCNG composites is highest among all samples (Figure 4c). It is worth pointing out that the UCNG composites deliver the outstanding rate capability. With current density increasing from 1 to 10 A g^−1^, the capacitance retention of the UCNG composites is 93.7%, much larger than the P‐Co_3_O_4_ (45.7%) and most of other reported Co_3_O_4_‐based electrodes and even superior to some advanced carbon materials for high‐rate supercapacitors (Tables S3 and S4, Supporting Information). The reason can be explained by the high electrical conductivity of the graphene sheets and the ultrafine Co_3_O_4_ nanoparticles. More importantly, the oxygen vacancies on the ultrafine Co_3_O_4_ nanoparticles surface in the UCNG composites induced by laser irradiation may play a key role in enhancing the rate capability. This result is confirmed by the fact that LP‐Co_3_O_4_ also shows high capacitance retention (85.8%) with increasing the current density from 1 to 10 A g^−1^.

The electrochemical performance of the UCNG composites was further investigated by using EIS (Figure 4d). The impedance characteristics were analyzed by the complex nonlinear least‐squares fitting method on the basis of a Randles equivalent circuit, as shown Figure S15 (Supporting Information). *R*_S_, *R*_CT_, *C*_DL_, *C*_F_, and *W*_o_ in the circuit represent solution resistance, charge‐transfer resistance, double‐layer capacitance, pseudocapacitance, and the finite‐length Warburg diffusion element, respectively. The impedance spectra can be classified into high and low frequency regions. The equivalent series resistance (ESR, 0.25 Ω) of the UCNG composites electrode is obtained from high‐frequency region of Nyquist plots (the inset of Figure 4d), which is much lower than the P‐Co_3_O_4_ and LP‐Co_3_O_4_ electrodes (ESR, 0.67 and 0.51 Ω), indicating a decreased electric resistance of the UCNG composites and interface resistance due to the incorporation of graphene. The enhanced conductivity of UCNG composites can be attributed to the combination of the unique synthesis procedures without using any surface modifying agent and organic additives, the plentiful oxygen vacancies created on the ultrafine Co_3_O_4_ nanoparticles surface, and the tightly anchoring of Co_3_O_4_ nanoparticles on the graphene. In the lower frequency region, the straight line was determined by ion diffusion. Obviously, the near‐zero slope for the UCNG composites and LP‐Co_3_O_4_ electrodes shows a lower diffusion resistance and an ideal polarizable capacitance in comparison with the P‐Co_3_O_4_ electrode, indicating an enhanced ion transmission properties from the laser‐induced effect (laser‐induced reduction and fragmentation). On the basis of above discussion, we might conclude that the combination of higher electrical conductivity and faster ion diffusion is responsible for the excellent rate capability of the UCNG composites.

**Figure 5:**
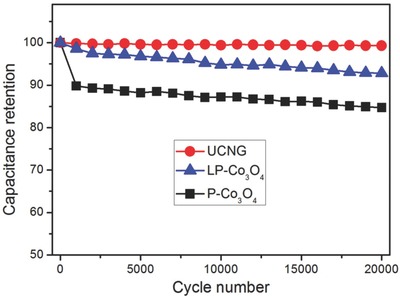
Cycle performances of the UCNG composites, P‐Co_3_O_4_, and LP‐Co_3_O_4_ electrodes over 20 000 cycles at a current density of 10 A g^−1^.

The cycle stability of the UCNG composites is investigated by repeating the charge/discharge test between −0.05 and 0.45 V at a high current density of 10 A g^−1^ for 20 000 cycles, which is a very significant requirement for the practical applications of supercapacitors. **Figure** **5** reveals that 99.3% of the initial capacitance is maintained after consecutive 20 000 cycles, demonstrating attractive stable cycling performance and a high level of reversibility of the UCNG composites. The morphology of the UCNG composites after 20 000 cycles (Figure S16, Supporting Information) is nearly unchanged, while the composition is still Co_3_O_4_ and graphene (Figure S17, Supporting Information). Besides, Figure S18 (Supporting Information) represents the Co 2p XPS spectrum for the UCNG composites after 20 000 cycles. Although the intensity of the satellite peaks related to the Co^2+^ oxidation state decreases, it still can be clearly observed, demonstrating that abundant oxygen vacancies are still retained on the ultrafine Co_3_O_4_ nanoparticles surface. These results further indicate the excellent cyclability of the UCNG composites. The cycle stability of the UCNG composites is remarkably superior to those of P‐Co_3_O_4_, and LP‐Co_3_O_4_ electrodes. After 20 000 cycles, the P‐Co_3_O_4_ and LP‐Co_3_O_4_ electrodes retain about 84.7% and 92.8% of its original capacitance, respectively. The enhanced cycle stability of the UCNG composites is mainly owing to the synergistic effect derived from ultrafine Co_3_O_4_ nanoparticles with surface oxygen vacancies and the graphene with high conductivity. As we know, a significant number of defect sites on the graphene surface induced by laser irradiation of GO can provide more active sites to anchor the Co_3_O_4_ nanoparticles and strengthen the binding energy between Co_3_O_4_ and graphene sheets, which leads to a more stable structure and a little loss of the capacitance during the cycling test. In addition, the UCNG exhibited high power density (4000 W kg^−1^) and energy density (43.1 Wh kg^−1^), as shown in Figure S19b (Supporting Information).

### 2.4 DFT Simulation

**Figure 6:**
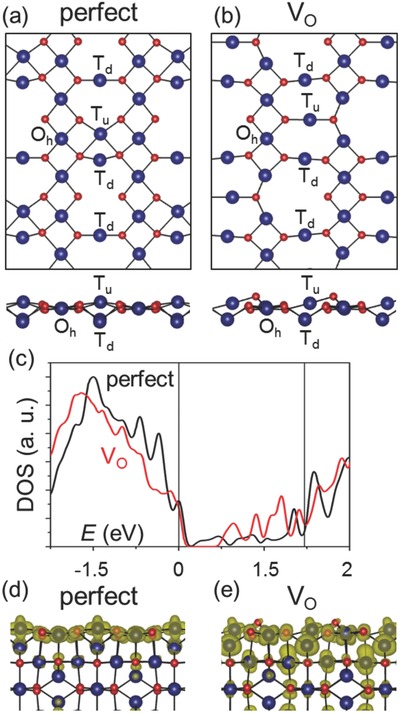
Atomic structure of Co_3_O_4_ (001) perfect a) and O deficient–*V*_O_ b) surface. Top panels show the top view and bottom panels show the side view. For clarity, only the atoms near the surface are shown. c) Electronic density of states of perfect (black) and *V*_O_ (red) surface. The vertical lines denote the band edge positions of the bulk Co_3_O_4_. Note that a Gaussian smearing of 0.05 eV is used for plotting. d,e) Charge density distribution of the states within the band gap of bulk Co_3_O_4_, for perfect d) and *V*_O_ e) surface.

To understand the origin of the oxygen vacancies (*V*_O_) enhanced performance, we perform spin‐polarized density functional theory (DFT) calculations with the local density approximation + Hubbard U correction (LDA+U) approach. The calculation details are shown in the Supporting Information. Co_3_O_4_ has a spinel structure, with Co^2+^ ions in tetrahedral interstices and Co^3+^ ions in the octahedral interstices of the cubic close‐packed lattice of oxide anions. We choose the (001) surface to study because it is the most common surface observed in experiments and has the lowest energy.^60^ Our calculations indicate that there are three types of Co atoms near the surface, as shown in **Figure** **6**a: One was in the octahedral sites of the bulk lattice but now is only bonded with 5 O atoms (Oh), one in the subsurface tetrahedral sites (Td), and the third was in the tetrahedral sites above the surface but now is bonded with 4 O atoms on the surface due to the reconstruction (Tu). We find that the low coordination induces magnetism at the Oh atoms, with a magnetic moment (μ) of ≈2 μ_B_. For comparison, the Co atoms in the bulk octahedral sites (Co^3+^) have μ = 0, while those in the tetrahedral sites (Co^2+^) has μ ≈ 2.6 μ_B_. The surface contributes new electronic states inside the band gap of bulk Co_3_O_4_, as shown in Figure 6c.

Figure 6b shows the lowest energy structure with O vacancies (*V*_O_). The *V*_O_ further reduces the coordination of some of the surface Oh Co atoms to from 5 to 4. This is accompanied by the change of μ to ≈2.6 μ_B_, similar to the Co^2+^ atoms in the bulk tetrahedral sites. Hence, the valance state of those Oh atoms is reduced to 2+, in agreement with the experiments. The *V*_O_ also reduces the coordination of some of the Co atoms in the subsurface octahedral sites from 6 to 5, enhancing their contribution to the band gap states (Figure 6d,e). As a result, the density of states within the band gap is significantly increased (Figure 6c). These states serve as electron reservoir during the electrochemical process, hence facilitate the charge transfer.^61^, ^62^ The defects enhanced electrochemical performance has also been observed in other systems such as batteries.^62^, ^63^, ^64^ Moreover, it has also been shown that *V*_O_ in the bulk can increase the electrical conductivity.^51^

From the above characterization results, it should be noted that UCNG composites would be highly suitable as a high‐rate electrode material for SCs, primarily due to the following factors. (i) Well‐dispersed ultrafine Co_3_O_4_ nanoparticles on the highly conductive graphene sheets can substantially improve the electrochemical utilization of Co_3_O_4_ and shorten the path lengths for both ion and electron transport. (ii) The oxygen vacancies by laser irradiation dramatically increase the active sites on the Co_3_O_4_ surface and the conductivity of Co_3_O_4_, which promotes the electrochemical activity and facilitates the electrochemical reaction. (iii) The defect sites created by the laser on graphene and the large surface area of graphene efficaciously prevent the aggregation of Co_3_O_4_ nanoparticles, thus enhancing the rate performance and cycling stability.

Researches for the generation of appropriate oxygen vacancies in metal oxide and the formation of reduced graphene oxide have long focused on traditional reductive method by reducing agent, reducing gas, etc.,^19^, ^20^, ^23^, ^24^, ^25^, ^26^, ^27^, ^28^, ^29^ respectively, the laser irradiation reduction route presented here opens the door to a new strategy producing oxygen vacancies in metal oxide and obtaining reduced graphene oxide, simultaneously. Moreover, the materials with oxygen vacancies possess high‐specific capacitance and rate capability. In addition, the laser irradiation reduction route seems to be universal with other metal oxides, such as Fe_2_O_3_ and MnO_2_. To demonstrate the generality of this laser synthesis method, MnO_2_/graphene and Fe_2_O_3_/graphene composites were also fabricated under laser irradiation (Figures S20, 21, Supporting Information). MnO_2_/graphene and Fe_2_O_3_/graphene composites fabricated under laser irradiation also exhibit excellent electrochemical performance, especially for high‐rate capability (Figure S22, Supporting Information).

## 3 Conclusion

In summary, we take the Co_3_O_4_/graphene composite as a typical example and have developed a universal in situ laser‐assisted strategy to grow ultrafine metal oxides/graphene composites via simple laser irradiation in solution at ambient conditions. Under laser irradiation, the GO is reduced to LG with a low transfer resistance for efficient charge storage and delivery, while the porous Co_3_O_4_ nanorods completely smash into ultrafine Co_3_O_4_ nanoparticles with short ionic diffusion distance for fast ion transport. The as‐obtained UCNG composites exhibit high‐specific capacitance and excellent cycling stability. In particular, when the current density is increased from 1 to 10 A g^−1^, the capacitance of UCNG composites still retains 93.7%, exhibiting outstanding rate capability. ESR measurements and DFT calculations also reveal that new oxygen vacancies defect states are generated after laser irradiation, which serve as electron reservoir during the electrochemical process. We demonstrate that the unique UCNG composite nanostructures with the abundant oxygen vacancies on the ultrafine Co_3_O_4_ nanoparticles surfaces and the tightly anchoring of Co_3_O_4_ nanoparticles onto the graphene sheets could provide good electrical conductivity, high electrochemical activity, and effective pathways for rapid ionic/electronic transport and fast reversible Faradaic reactions. Combining the remarkable rate capability, outstanding capacitance and excellent cycle stability, we conclude that the UCNG composites are good electrode materials for supercapacitor compared to previous samples in literature. In addition, this novel synthetic method can be extended to other metal oxide/graphene composites, which will have great potentials in not only the energy storage field, but also in numerous other frontiers.

## 4 Experimental Section

*Synthesis of Porous Co_3_O_4_ Nanorods (P‐Co_3_O_4_)*: All chemicals were of analytical grade and used without further purification. In a typical synthesis, 0.996 g of cobalt acetate tetrahydrate (Co(CH_3_COO)_2_·4H_2_O) and 1.0 g of polyvinyl‐pyrrolidone (PVP K‐30) were added in 40 mL of polyethylene glycol (PEG‐400). The mixture was kept vigorous stirring at 60 °C until Co(CH_3_COO)_2_·4H_2_O and PVP K‐30 were completely dissolved. Then, 1.0 g of urea (CO(NH_2_)_2_) was added to the resultant homogeneous solution. After stirring for 15 min, the reaction mixture from the first step was transferred to a Teflon‐lined stainless autoclave for solvothermal reaction at 160 °C for 20 h. After that, the product was washed with deionized water and ethanol for several times, and then dried in a vacuum oven at 60 °C for 12 h. The as‐prepared powder was annealed at 300 °C for 2 h in air with a heating rate of 1 °C min^−1^ to acquire porous Co_3_O_4_ nanorods (denoted as P‐Co_3_O_4_).

*Synthesis of UCNG Composites*: GO was synthesized from natural flake graphite (Alfa Aesar, 325 mesh, 99.8%) according to the modified Hummer's method.^19^ The preformed porous Co_3_O_4_ nanorods (0.25 g) were dispersed into 10 mL of GO solution (1.5 mg mL^−1^). Then the mixture solution was irradiated with the beam of a KrF excimer laser (10 Hz, 25 ns, Coherent, CompexPro 205) under a continuous magnetic stirring. The pulsed laser beam was guided by a reflection mirror and focused by a quartz lens on the mixture solution. The laser fluence was measured by a laser FieldMaxII Meter (Coherent). The optimal laser parameters are selected as following: 400 mJ pulse^−1^ cm^−2^ of laser fluence and 30 min of irradiating time. After laser irradiation, the products were collected by freeze drying for further characterization. The yield of ultrafine Co_3_O_4_ nanoparticles/graphene (UCNG) composites is about 94.3%. The maximal experimental throughput is ≈0.5 g h^−1^ for this typical ns laser system. For comparison, the laser‐reduced graphene was synthesized under the same experimental conditions in the absence of Co_3_O_4_ nanorods. Also, LP‐Co_3_O_4_ were also obtained in the absence of GO for controlled SCs measurement under similar laser conditions as described above.

*Characterization*: UV–vis absorption spectra were recorded on a Shimadzu UV‐3600 spectrophotometer in the 200–1000 nm wavelength range at room temperature. The powder XRD measurements were performed using a Bruker D8‐Advance diffractometer with Cu Kα radiation (λ = 1.5418 Å). The powder samples were mounted flat and scanned over the range 10°–80°. The microstructures of the samples were observed by SEM (FEI Quanta 250 FEG) and TEM (JEOL JEM‐2010F). Raman spectra were taken on a Thermo Scientific Raman Microscope DXR with a 532 nm laser excitation length. The XPS was measured on a Kratos AXIS Ultra DLD spectrometer with a monochromatic AlKa X‐ray source. The EPR spectra were obtained using a Bruker A300 EPR spectrometer at X‐band (≈9 GHz) at room temperatures. FTIR spectra were recorded using a Nicolet 6700 FTIR spectrometer.

*Electrochemical Measurements*: All electrochemical measurements were performed on a Zahner/Zennium electrochemical workstation at room temperature. The working electrodes were fabricated as follows. Briefly, the as‐prepared composites, acetylene black, and polyvinylidene difluoride were mixed in a mass ratio of 85:10:5 and dispersed in *N*‐methyl pyrrolidone solvent. Then, the slurry was pasted on the nickel foam substrate (1 cm × 1 cm) with a spatula and dried at 60 °C for 24 h. Finally, the electrode plate was pressed under a pressure of 10 MPa. The mass of active materials of (ultrafine Co_3_O_4_ nanoparticles/graphene composites) on current collector (nickel foam) was 6.5 mg cm^−2^. The monolithic electrode was tested in three‐electrode configuration assembled with a platinum foil (1 cm × 1 cm) auxiliary electrode and the Ag/AgCl reference electrode by CV, GCD, and EIS in an aqueous KOH electrolyte (2.0 m). The specific capacitance of electrode material was calculated from the charge and discharge curves, according to *C = IΔt/ΔVm*, where *C* is the specific capacitance (F g^−1^), *Δt* is the discharge time (s), *I* is the discharge current (A), *ΔV* is the operating potential window (*V*) during the discharge, and *m* is the mass (g) of active materials (UCNG composites) in as‐prepared electrode. EIS measurements were conducted with an AC amplitude of 5 mV in the 100 mHz–100 kHz frequency range.

*Spin‐Polarized DFT Computation*: Spin‐polarized DFT calculations were performed using the Vienna Ab‐initio Simulation Package (VASP)^65^, ^66^ with projector augmented wave pseudopotentials^67^, ^68^ and the Perdew–Burke–Ernzerhof exchange‐correlation functional.^69^ The electron correlation was remedied by using the LDA+U approach,^70^ with *U* = 3 eV for Co d‐electrons, which has been shown to reproduce well the experimental structural parameters, heat of formation, and the band gap.^71^ The atomic structures and the coordinates (in VASP CONTCAR format) used to model the 001 surface are shown in Figure S23 (Supporting Information). We used 400 eV for the plane‐wave cutoff and fully relaxed the systems until the final force on each atom is less than 0.01 eV Å^−1^. 5 × 5 × 1 k‐points with Monkhorst–Pack sampling^72^ are used to relax the systems, and 21 × 21 × 1 k‐points are used to calculate the density of state (DOS).

## Conflict of Interest

The authors declare no conflict of interest.

## Supporting information

Supplementary
