# Supplementary material for: Oxygen‐Vacancy Abundant Ultrafine Co3O4/Graphene Composites for High‐Rate Supercapacitor Electrodes
Source: Adv Sci (Weinh). 2018 Jan 15;5(4):1700659. doi: 10.1002/advs.201700659 (PMC5908357; doi:10.1002/advs.201700659)
Supplement: Supplementary file 1 — Supplementary [file ADVS-5-1700659-s001.pdf]

Copyright WILEY-VCH Verlag GmbH & Co. KGaA, 69469 Weinheim, Germany, 2017.

ADVANCED SCIENCE Open Access

Supporting Information

for *Adv. Sci.,* DOI: 10.1002/advs.201700659

Oxygen-Vacancy Abundant Ultrafine Co_3O_4/Graphene Composites for High-Rate Supercapacitor Electrodes

Shuhua Yang, Yuanyue Liu, Yufeng Hao, Xiaopeng Yang, William A. Goddard, Xiao Li Zhang, and Bingqiang Cao**

Copyright WILEY-VCH Verlag GmbH & Co. KGaA, 69469 Weinheim, Germany, 2013.

Supporting Information

Oxygen-Vacancy Abundant Ultrafine Co_3O_4/Graphene Composites for High-Rate Supercapacitor Electrodes

Shuhua Yang, Yuanyue Liu, Yufeng Hao, Xiaopeng Yang, William A. Goddard III, Xiao Li Zhang, and Bingqiang Cao**

Dr. S. H. Yang, Dr. X. P. Yang, Prof. Dr. B. Q. Cao
Materials Center for Energy and Photoelectrochemical Conversion, School of Material Science and Engineering
University of Jinan
Jinan 250022, China
E-mail: mse_caobq@ujn.edu.cn (B. Q. Cao)
Dr. Y. Y. Liu, Prof. W. A. Goddard III
Materials and Process Simulation Center
California Institute of Technology
Pasadena, CA 91125, US
Prof. Dr. Y. F. Hao
National Laboratory of Solid State Microstructures, College of Engineering and Applied Sciences, and Collaborative Innovation Center of Advanced Microstructures
Nanjing University
Nanjing 210093, China
Dr. Y. Y. Liu
The Resnick Sustainability Institute
California Institute of Technology
Pasadena, CA 91125, US
E-mail: yuanyue.liu.microman@gmail.com (Y. Y. Liu)
Prof. Dr. X. L. Zhang
School of Materials Science and Engineering, and State Centre for International Cooperation on Designer Low-Carbon & Environmental Materials
Zhengzhou University
Zhengzhou 450001, China

Keywords: ultrafine Co_3O_4 nanoparticles, graphene, laser irradiation, oxygen vacancies, supercapacitors

Part I: Supplementary Figures and Tables

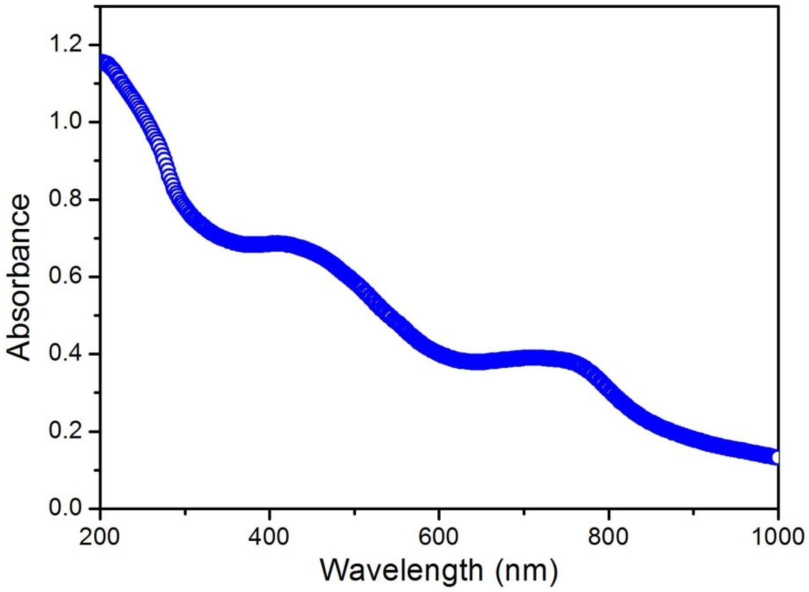

Figure S1 UV-vis absorption spectrum of the porous Co_3O_4 nanorods.

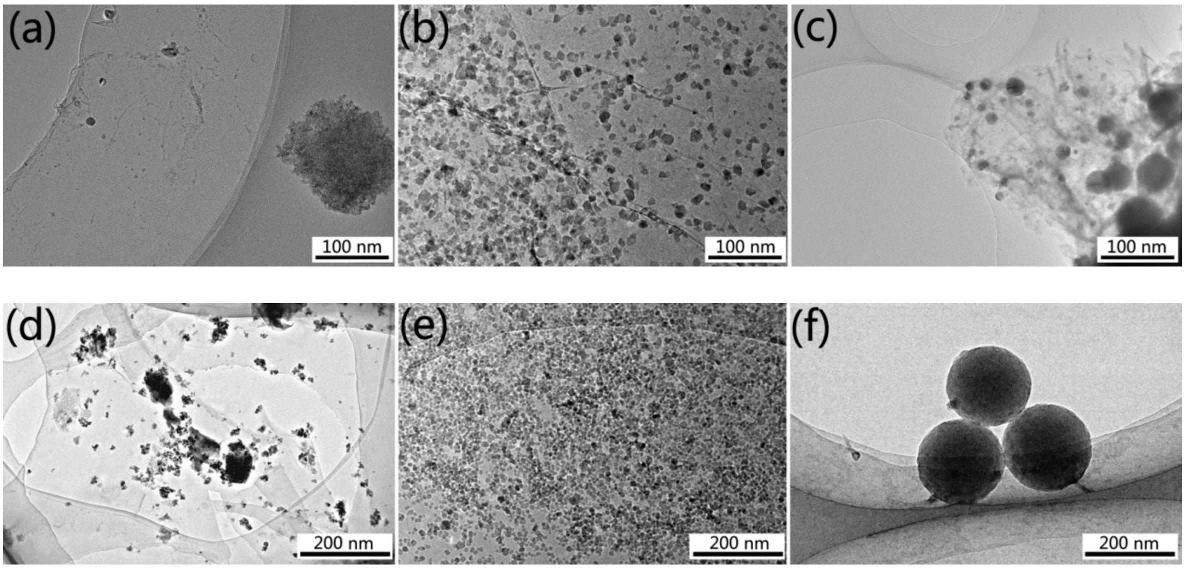

Figure S2 TEM images of UCNG-10 (a), UCNG (b), UCNG-60 (c) under the same laser fluence (400 mJ pulse^{-1} cm^{-2}) with different irradiation time (10, 30, 60 min); TEM images of UCNG-200 (d), UCNG (e), and UCNG-600 (f) under the same irradiation time (30 min) with different laser fluence (200, 400, 600 mJ pulse^{-1} cm^{-2}). The figure S2b and the figure S2e are from the same sample (UCNG), but the scales are different.

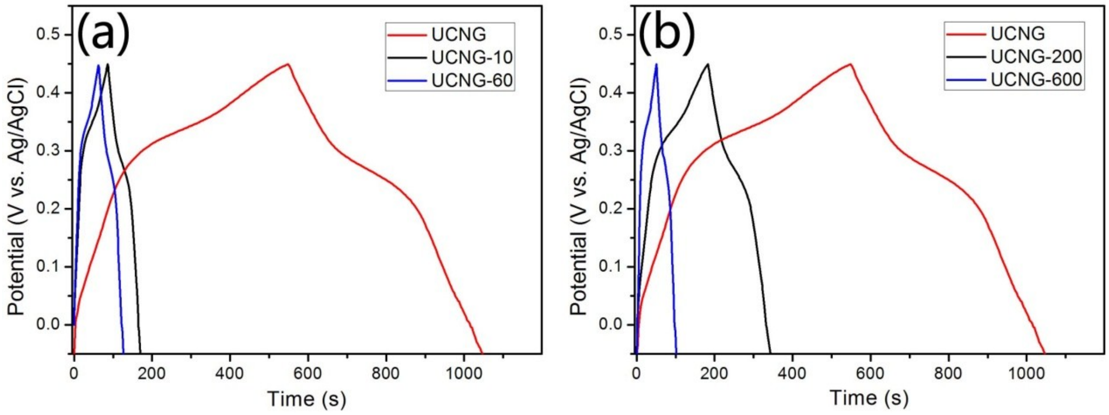

Figure S3 (a) GCD curves of the Co_3O_4 nanoparticles/graphene composites under same laser fluence with different irradiation time, (b) GCD curves of the Co_3O_4 nanoparticles/graphene composites under same irradiation time with different laser fluence. The current density is 1 Ag^{-1}.

Table S1 Various Co_3O_4 nanoparticles/graphene composites prepared under different laser parameters.

Conditions	10 min	30 min	60 min
**200 mJ pulse^{-1} cm^{-2}**	N/A	UCNG-200	N/A
**400 mJ pulse^{-1} cm^{-2}**	UCNG-10	UCNG	UCNG-60
**600 mJ pulse^{-1} cm^{-2}**	N/A	UCNG-600	N/A

Table S2 The capacitance (capacity) of various Co_3O_4 nanoparticles/graphene composites prepared under different laser parameters.

Materials	UCNG-10	UCNG-60	UCNG	UCNG-200	UCNG-600
**Capacitance (F/g)**	167.4	127.3	978.1	320.5	101.2
**Capacity (mAh/g)**	23.2	17.7	135.8	44.5	14.1

A number of Co_3O_4 nanoparticles/graphene composites prepared under various laser parameters (Table S1) were surveyed to prove the proposed strategy in paper and optimize the preparation condition associated with excellent electrochemical properties for SCs electrodes applications.

Figure S2a-c reveals the morphology evolution of Co_3O_4 nanoparticles/graphene composites with different irradiation time (10, 30, 60 min) under the same laser fluence (400 mJ pulse^{-1} cm^{-2}). When the laser irradiation time is as short as 10 min, the porous Co_3O_4 nanorods have been fragmented into ultrafine Co_3O_4 nanoparticles, but only few particles are anchored on LG surface, as shown in Figure S2a. This is because the laser energy is enough to fragment the porous Co_3O_4 nanorods, but the laser irradiation time is too short to disperse and anchor the ultrafine Co_3O_4 nanoparticles on LG surface. When laser irradiation time increase to 30 min, all ultrafine Co_3O_4 nanoparticles are well dispersed on LG surface and no particles outside LG surface are found (Figure S2b). When the laser irradiation time further increase (60 min), the ultrafine Co_3O_4 nanoparticles on LG surface fuse together and form bigger spherical particles, as shown in Figure S2c.

Figure S2d-f reveals the morphology evolution of Co_3O_4 nanoparticles/graphene composites with different laser fluence (200, 400, 600 mJ pulse^{-1} cm^{-2}) for the same

irradiation time (30 min). As the applied laser energy density is 200 mJ pulse^{-1} cm^{-2}, it is too low to fragment the porous Co_3O_4 nanorods, as shown in Figure S2d. When the laser increase to 400 mJ pulse^{-1} cm^{-2}, the porous Co_3O_4 nanorod are completely fragmented into ultrafine Co_3O_4 nanoparticles, as shown in Figure S2e. The ultrafine Co_3O_4 nanoparticles merge and form bigger spherical particles under 600 mJ pulse^{-1} cm^{-2} laser energy density, which is high enough to melt the particles (Figure S2f). This indicates that lasers with proper irradiation time and fluence (400 mJ pulse^{-1} cm^{-2} of laser fluence and 30 min of irradiating time) are necessary to prepare ultrafine Co_3O_4 nanoparticles/graphene composites.

Figure S3 shows the galvanostatic charge/discharge (GCD) curves of the various Co_3O_4 nanoparticles/graphene composites at 1 A g^{-1}. Among various Co_3O_4 nanoparticles/graphene composites, the UCNG composites exhibit the highest specific capacitance (Table S2), further confirming the optimized preparation condition (400 mJ pulse^{-1} cm^{-2} of laser fluence and 30 min of irradiating time).

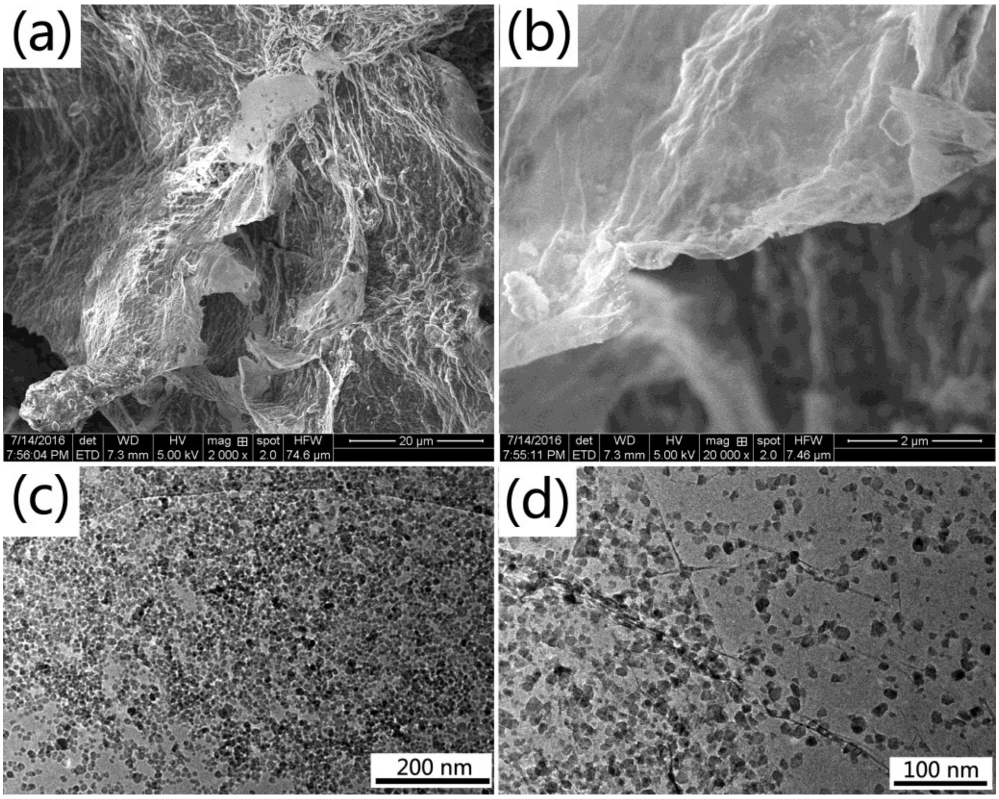

Figure S4 (a) SEM image, (b) enlarged SEM image, (c) TEM image, and (d) enlarged TEM image of the UCNG composites.

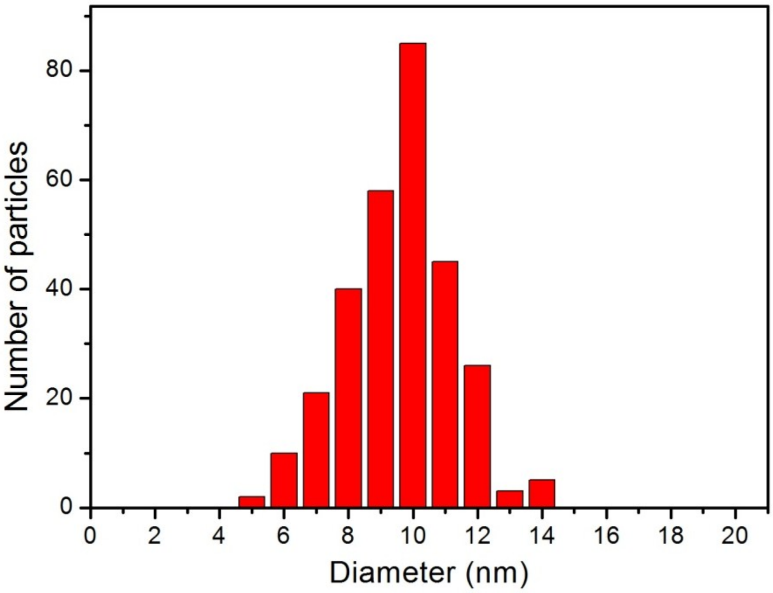

Figure S5 Size distribution of Co_3O_4 nanoparticles in the UCNG composites.

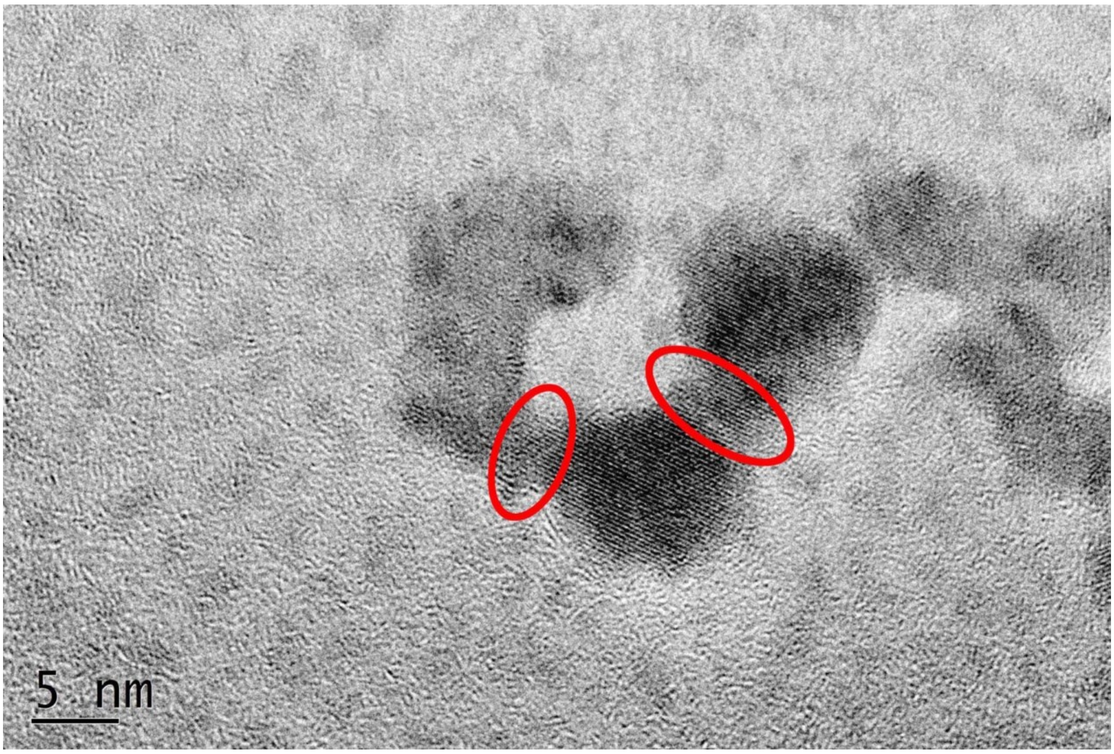

Figure S6 (a, b) TEM image of ultrafine Co_3O_4 nanoparticles on graphene sheets after laser irradiation. The neck formation (slight fusion) between adjacent Co_3O_4 particles may be mainly caused by laser melting. The neck was indicated by the red circle.

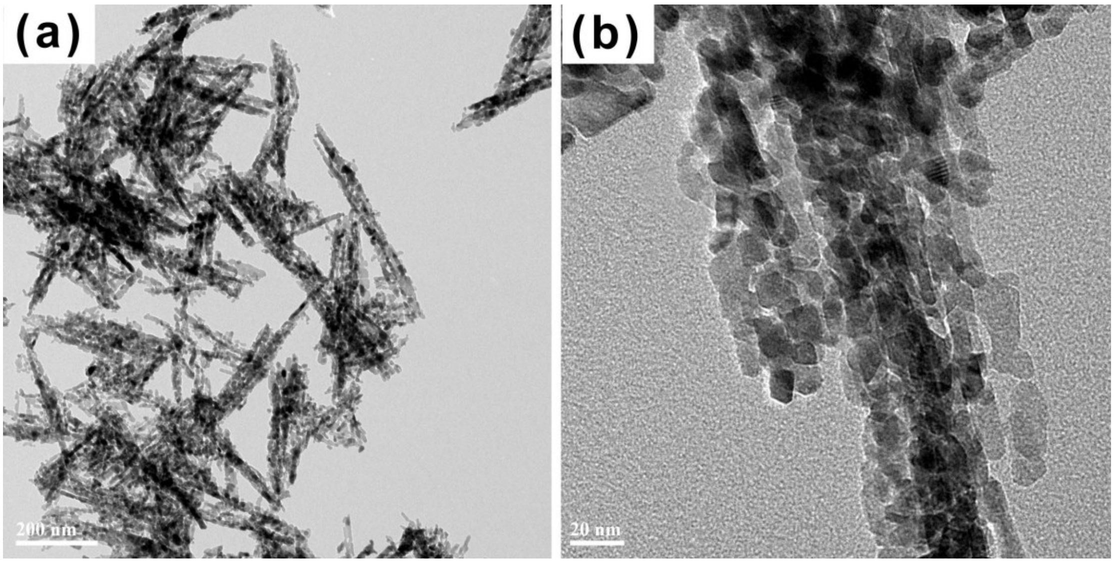

Figure S7 (a) TEM image and (b) zoom-in TEM image of porous Co_3O_4 nanorods (P-Co_3O_4) before laser irradiation.

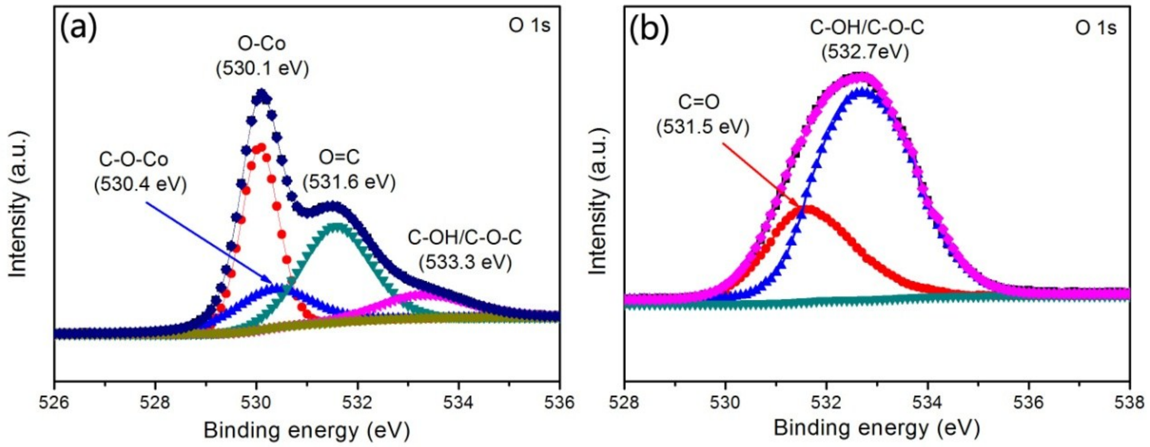

Figure S8 High-resolution O1s XPS spectra of LG (a) and UCNG composites (b).

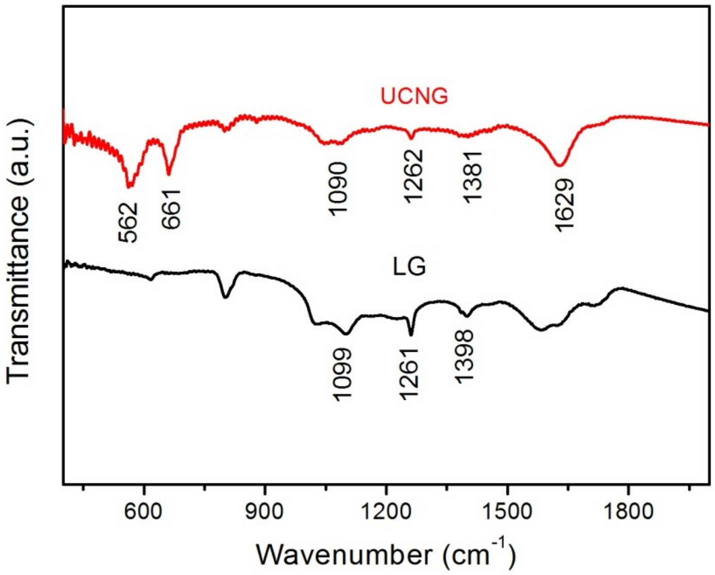

Figure S9 FTIR spectra of LG (a) and UCNG composites (b).

The interaction between graphene and Co_3O_4 nanoparticles is further corroborated by XPS and Fourier transform infrared (FTIR) spectroscopy (Figure S8, S9).

High-resolution O1s XPS spectra was obtained in the LG (laser reduced graphene) and ultrafine Co_3O_4 nanoparticles/graphene (UCNG) composites. The O1s XPS spectrum of UCNG can be deconvoluted into four peaks (Figure S8a). The peak located at 530.1 is attributed to O-Co bonding configuration in Co_3O_4.[1] The peak at 531.6 eV is related to C=O bonding configuration while the peak at 533.3 eV is due to the C-OH and/or C-O-C bonding

configuration.[2, 3] Another peak centered at 530.4 eV originates from the possible formation of a Co-O-C bond, exhibiting that Co_3O_4 was anchored on the graphene sheets by a Co-O-C.[2, 4] Comparing with the peaks at binding energies of 531.5 and 532.7 eV in O 1s XPS spectrum of the LG (Figure S8b), the intensities of the O 1s peaks associated with C=O group and C-OH and/or C-O-C group in UCNG decreased dramatically, indicating that the oxygen-containing functional groups on LG have been substituted by cobalt ion in Co_3O_4, forming the Co-O-C bonds.

The FTIR spectra of the LG (laser reduced graphene) and ultrafine Co_3O_4 nanoparticles/graphene (UCNG) composites in the 400-2000 cm^{-1} spectral region were shown in Figure S9. In the FTIR spectrum of the UCNG, the peaks at 562 and 661 cm^{-1} are derived from the characteristic Co-O vibrations of the Co_3O_4 spinel lattice.[5] The FTIR spectrum of LG shows the characteristic peaks at 1398, 1261, and 1099 cm^{-1} are attributed to O-H deformation vibration, C-O vibration of the carboxy, and C-O-C vibration of the epoxy, respectively.[2, 6] However, after the growth of Co_3O_4 nanoparticles, the peaks corresponding to the O-H and C-O-C groups at 1398 and 1099 cm^{-1} almost disappear, while the intensity of C-OH group at 1261 cm^{-1} significantly decrease. This result suggests that the epoxy C-O and hydroxyl O-H groups have been broken down to form a Co-O-C bond between graphene and Co_3O_4 nanoparticles.[4]

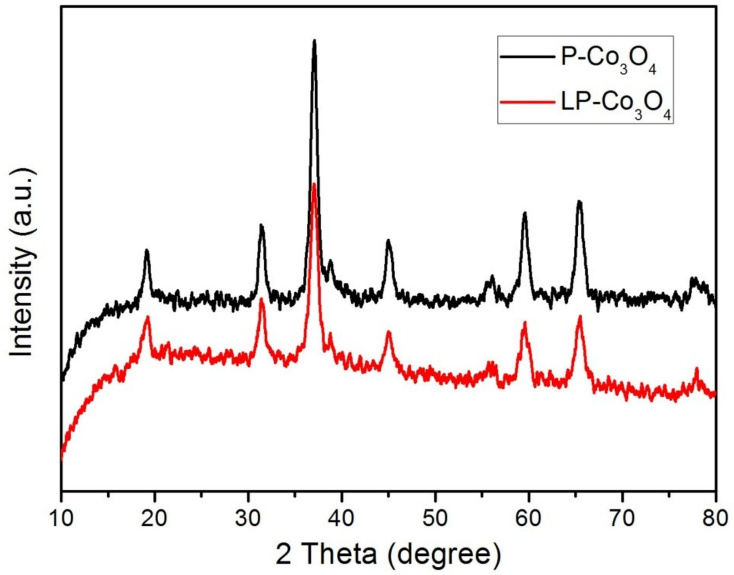

Figure S10 XRD diffractograms of P-Co_3O_4 and LP-Co_3O_4.

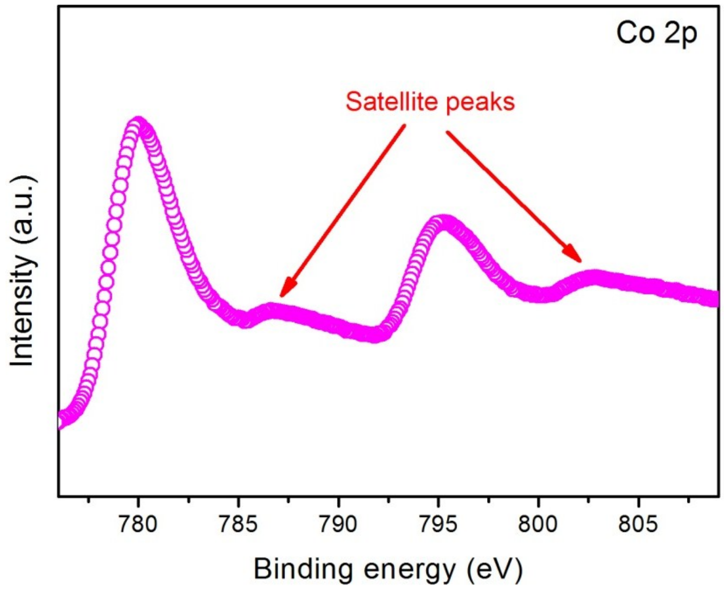

Figure S11 Co 2p XPS spectrum for LP-Co_3O_4.

In the high-resolution Co 2p spectra (Figure 3b and Figure S11), the peaks observed at 780.0 and 795.5 eV for the P-Co_3O_4 (780.0 and 795.3 eV for the LP-Co_3O_4) can be assigned to the Co $2p_{3/2}$ and Co $2p_{1/2}$ spin-orbital peaks of Co_3O_4, respectively. Compared to the P-Co_3O_4, the Co 2p peaks of the LP-Co_3O_4 show two more obvious satellite peaks centered at about 786.6 and 802.8 eV, which are attributed to the Co^{2+} oxidation state, indicating that a part of the Co^{3+} ions was reduecd to Co^{2+} and formed oxygen vacancies.

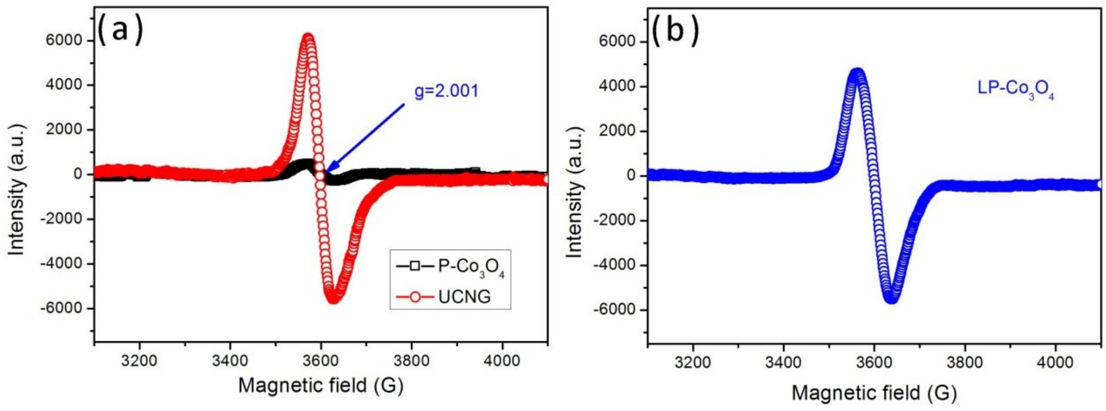

Figure S12 (a) Room-temperature EPR spectra of UCNG composites and P-Co_3O_4. (b) Room-temperature EPR spectrum of LP-Co_3O_4. The signal intensity illustrates that both UCNG composites and LP-Co_3O_4 obtained under the laser irradiation possess higher oxygen vacancy concentration, while the P-Co_3O_4 exhibit very low oxygen vacancy concentrations, which agrees well with the XPS results.

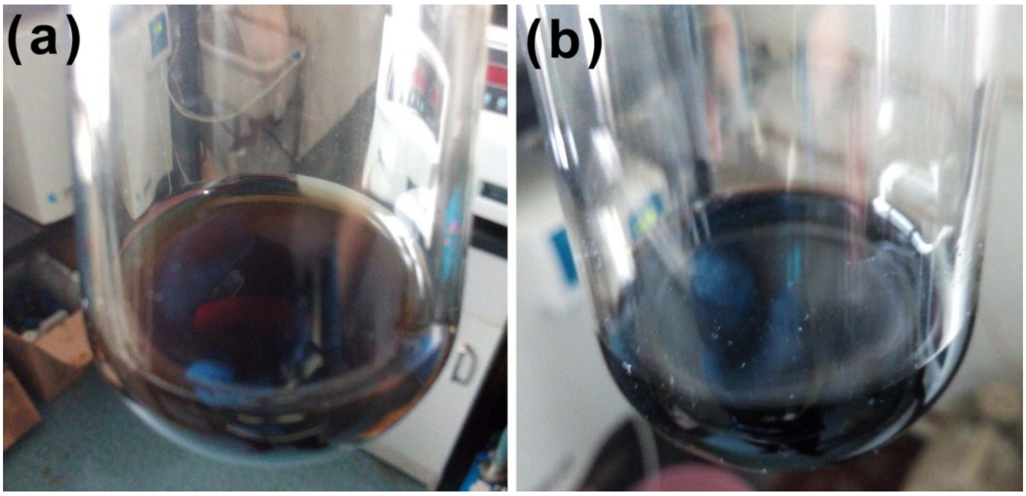

Figure S13 Optical images of the mixture solution for GO and porous Co_3O_4 nanorods before (a) and after (b) laser irradiation. Upon laser irradiation, the yellow–brown color instantaneously turned black, indicating that GO could be rapidly reduced by laser irradiation.

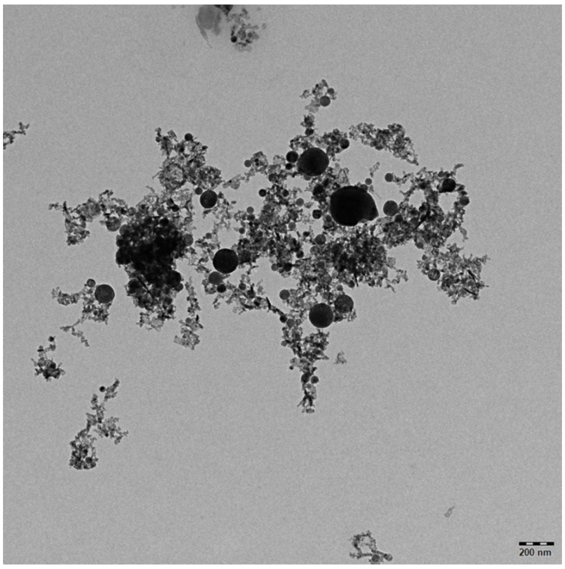

Figure S14 TEM image of LP-Co_3O_4. In the absence of GO, the LP-Co_3O_4 was prepared under same laser conditions as described in EXPERIMENTAL SECTION.

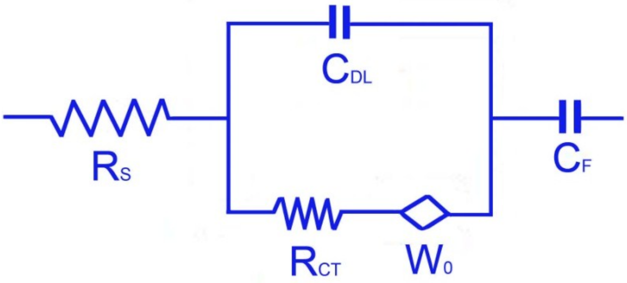

Figure S15 Equivalent circuit for three-electrode configuration cell used in this work. The impedance characteristics were analyzed by the complex nonlinear least-squares (CNLS) fitting method on the basis of a Randles equivalent circuit, as shown Figure S15 (Supporting Information). R_S, R_{CT}, C_{DL}, C_F, and W_o in the circuit represents solution resistance, charge-transfer resistance, double-layer capacitance, pseudocapacitance, and the finite-length Warburg diffusion element, respectively.

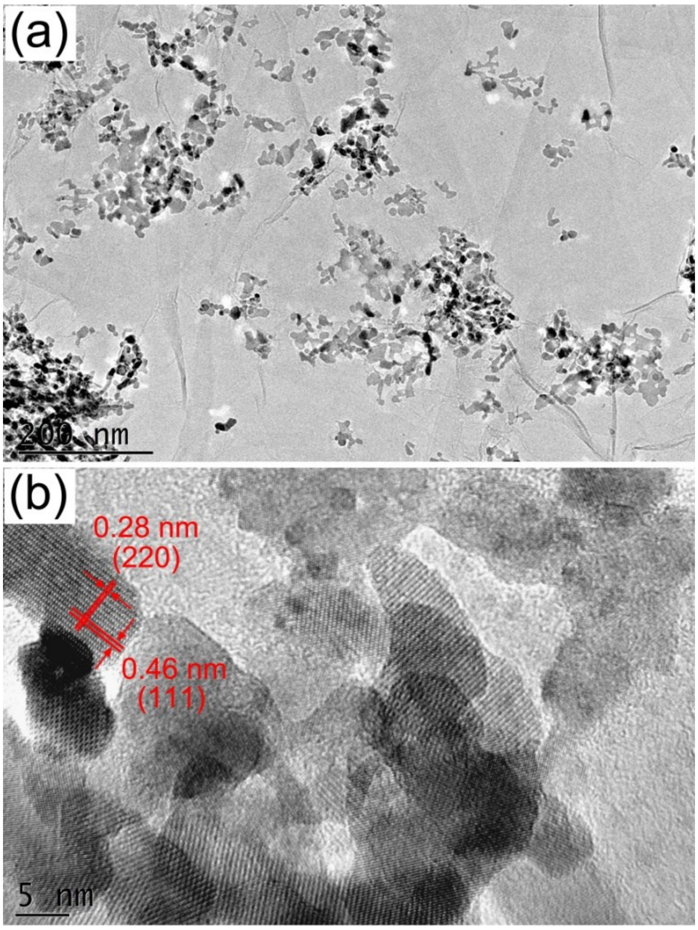

Figure S16 (a) TEM image and (b) HRTEM image of the UCNG composites after 20000 cycles.

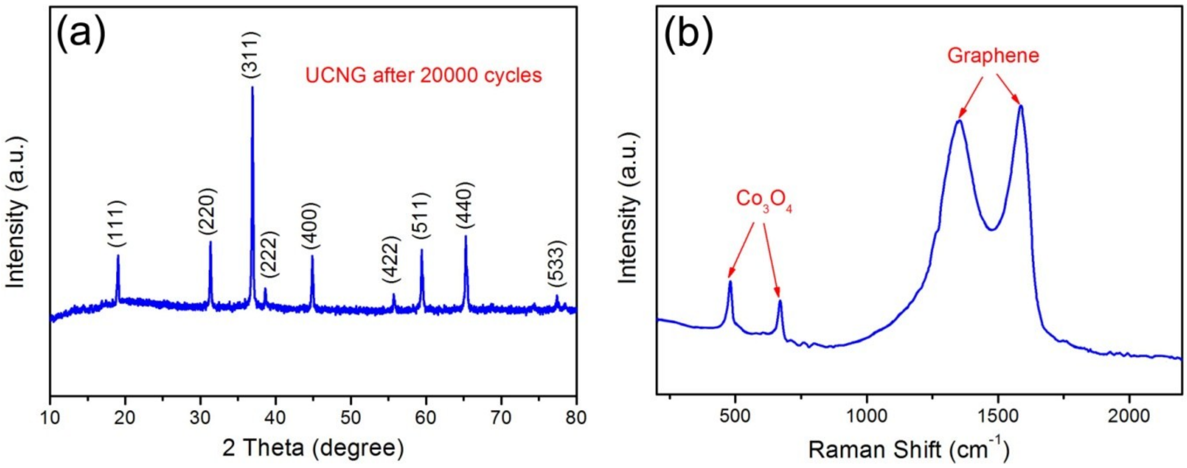

Figure S17 (a) XRD diffractogram and (b) Raman spectrum of the UCNG composites after 20000 cycles.

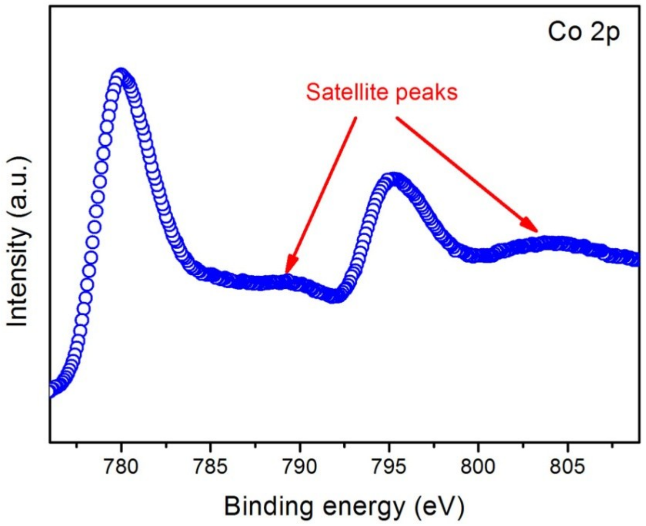

Figure S18 Co 2p XPS spectrum for the UCNG composites after 20000 cycles.

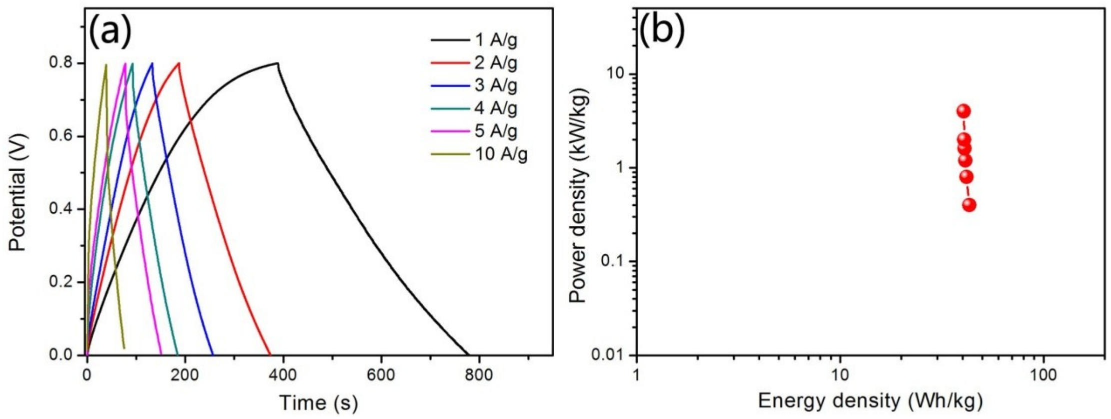

Figure S19 (a) GCD curves at different current densities, (b) Ragone plot (power density vs energy density) of symmetric supercapacitors based on UCNG.

The power density (P) and energy density (E) of the ultrafine Co_3O_4 nanoparticles/graphene composites (UCNG) were tested by a classical two-electrode configuration in 2 M KOH aqueous electrolyte.

Specific capacitance of the UCNG in the symmetric supercapacitors (two-electrode configuration) were calculated from the galvanostatic charge–discharge (GCD) curves according to Eq. (1)

$$C=2I\Delta t/\Delta Vm \qquad (1)$$

where C is the specific capacitance (F g^{-1}), I is the current (A), Δt is the discharge time (s), m is the mass of one electrode (g), and ΔV is the operating potential window (V) during the discharge.

The potential range was set between 0 and 0.8 V.

The energy density (E) and power density (P) of the UCNG were calculated by using the following equations:

$$E = 0.5C\,(\Delta U)^2/3.6 \quad (2)$$

$$P = E/\Delta t \quad (3)$$

where E (Wh/kg) is the energy density, C (F/g) is the specific capacitance, ΔU (V) was the SCs voltage window, P (W/kg) is the power density, and Δt (h) is the discharge time.

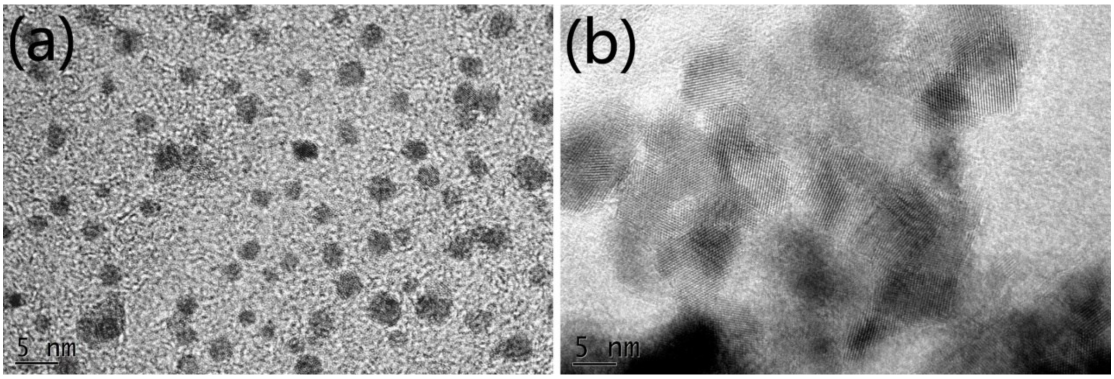

Figure S20 TEM images of (a) Fe_2O_3/graphene and (b) MnO_2/graphene composites. After laser irradiation, the Fe_2O_3 and MnO_2 precursors completely break up into ultrafine Fe_2O_3 and MnO_2 nanoparticles, and the ultrafine Fe_2O_3 nanoparticles and ultrafine MnO_2 nanoparticles were simultaneously anchored on graphene sheets.

For comparison, Fe_2O_3 and MnO_2 samples were prepared as given in previous reports (*Chem. Commun.* **2002**, 764, *Adv. Funct. Mater.* **2013**, 23, 4049). L-Fe_2O_3 and L-MnO_2 (Fe_2O_3/graphene and MnO_2/graphene composites) were fabricated using the as-prepared Fe_2O_3 and MnO_2 samples by laser irradiation reduction route.

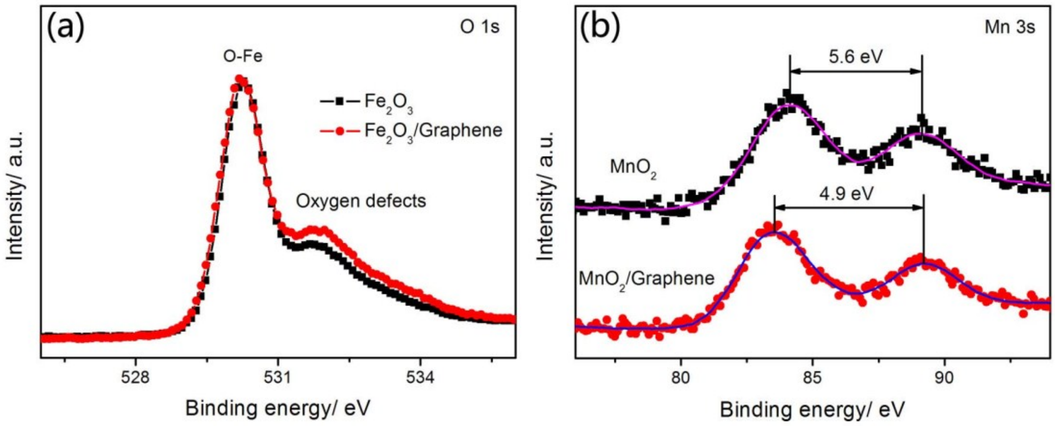

Figure S21 (a) Narrow O 1s XPS spectra collected for untreated Fe_2O_3 and Fe_2O_3/graphene samples, (b) narrow Mn 3s XPS spectra collected for untreated MnO_2 and MnO_2/graphene samples.

For the O 1s XPS spectra of the untreated Fe_2O_3 and Fe_2O_3/graphene samples, two peaks can be obviously identified for both samples. The peak centered at about 530.2 eV is attributed to O-Fe bonding configuration in Fe_2O_3, while the peak located at 531.7 eV is related to the oxygen defects in the matrix of metal oxides (*Adv. Mater.* **2014**, 26, 3148). In comparison with the untreated Fe_2O_3 sample, the higher intensity of the peak located at 531.7 eV for Fe_2O_3/graphene sample indicates that Fe_2O_3 in Fe_2O_3/graphene sample has more oxygen defects. For the Mn 3s XPS spectra of the untreated MnO_2 and MnO_2/graphene samples, the MnO_2/graphene sample shows larger energy separation (ΔE=5.6 eV) when compared with untreated MnO_2 sample (ΔE=4.9 eV). As previously reported (*Nano Energy* **2014**, 8, 255), the multiplet splitting energy (ΔE) increases linearly as the valence of Mn element decreases. This result further confirms that the MnO_2 in MnO_2/graphene sample is reduced by laser irradiation (Mn^{4+} to Mn^{3+}/Mn^{2+}) and plentiful oxygen vacancies are induced into MnO_2.

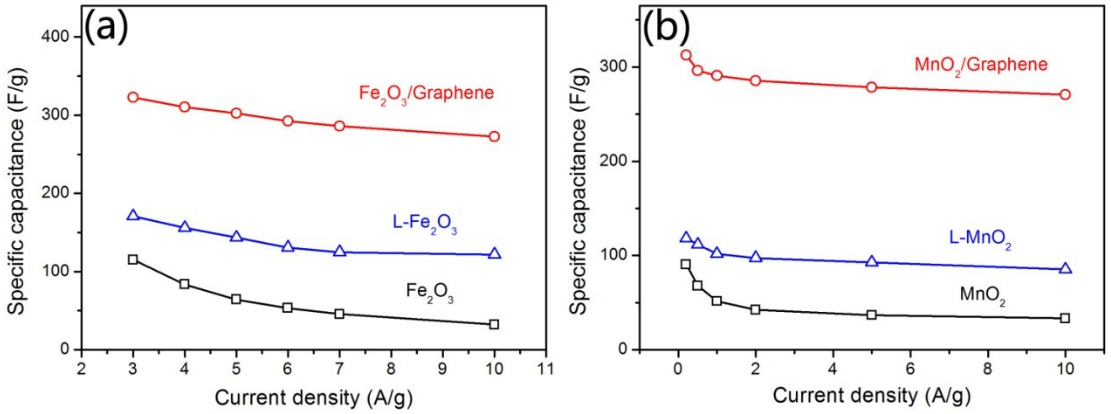

Figure S22 (a) Specific capacitance of Fe_2O_3/graphene composites, Fe_2O_3, and L-Fe_2O_3 electrodes calculated from GCD curves as a function of current densities, (b) Specific capacitance of MnO_2/graphene composites, MnO_2, and L-MnO_2 electrodes calculated from GCD curves as a function of current densities.

The electrochemical measurements of Fe_2O_3 based electrodes were tested using a three-electrode cell in 1 M Na_2SO_4 aqueous solution within a potential window from -1 to -0.3 V (vs. Ag/AgCl). The electrochemical measurements of MnO_2 based electrodes were tested using a three-electrode cell in 1 M Na_2SO_4 aqueous solution within a potential window from 0 to 1 V (vs. Ag/AgCl).

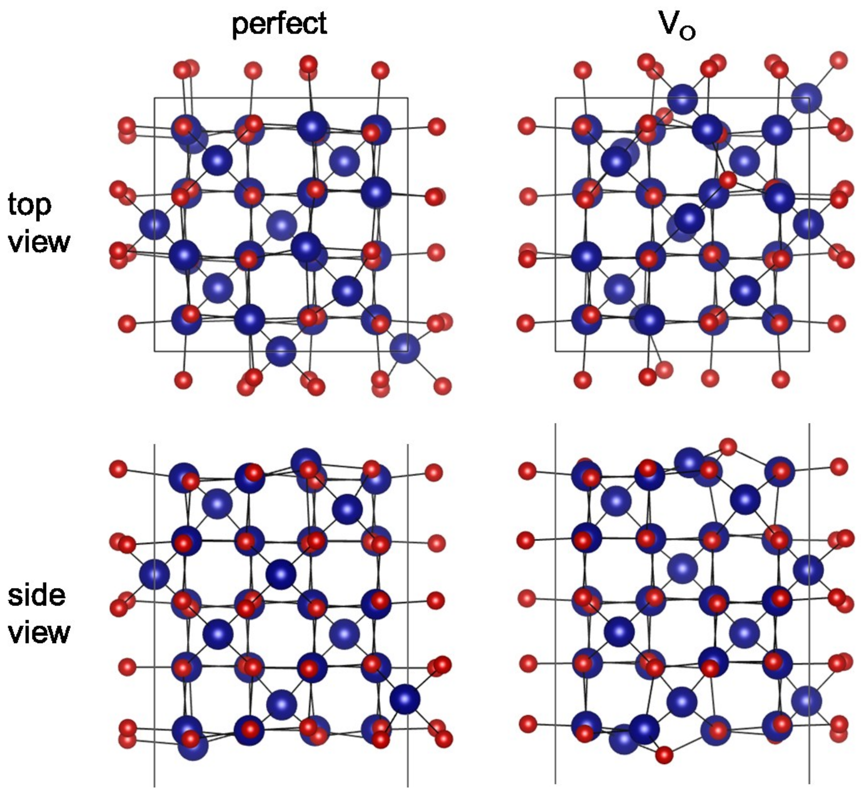

Figure S23 Atomic structures used to model the 001 surface

Table S3 Comparison of the rate capability of other Co_3O_4-based electrodes in the literature.

Materials	Electrolyte	Measurement methods	C_s/ F g^{-1} (I_s / A g^{-1})	Rate capability	Ref.
**Mesoporous Co_3O_4 nanosheet**	2 M KOH	3-electrode	2735 (2)<br>1471 (10)	53.8%	[7]
**3D Graphene-Based Aerogel/ Co_3O_4**	6 M KOH<br>6 M KOH	3-electrode<br>2-electrode	660 (0.5)<br>500 (10)<br>130 (0.455)<br>103 (9.09)	75.8%<br>79.2%	[8]
**Co_3O_4/C-800**	2 M KOH	3-electrode	201 (1)<br>176.9 (20)	88%	[9]
**RGO-Co_3O_4**	2 M KOH	3-electrode	458 (0.5)<br>416 (2)	90.8%	[10]
**3D Co_3O_4 twin-spheres**	6 M KOH	3-electrode	781 (0.5)<br>611 (8)	78.2 %	[11]
**Reduced Mesoporous Co_3O_4 Nanowires**	1 M KOH	3-electrode	977 (2)<br>484 (10)	49.5%	[12]
**Co_3O_4/carbon**	6 M KOH	3-electrode	555. 2 (1)<br>411. 8 (40)	74.2%	[13]
**CNT@ Co_3O_4**	PVA-H_2SO_4 gel	2-electrode	~90 (~0.1)<br>~30 (~1.9)	33.3%	[14]
**Sub−3 nm Co_3O_4 Nanofilms**	2 M KOH	3-electrode	1400 (1)<br>1276 (8)	91.1%	[15]
**Co_3O_4 / Vertically aligned graphene**	PVA/KOH gel	2-electrode	580 (1)<br>300 (10)<br>196 (40)	51.7%<br>33.8%	[16]
**Mesoporous Co_3O_4 Microtubules**	3 M KOH	3-electrode	130.5 (0.5)<br>119.0 (2)	91.2%	[17]
**Needle-like Co_3O_4 /Graphene**	2 M KOH	3-electrode	157.7 (0.1)<br>60 (2)	38.0%	[18]
**Co_3O_4 nanosheets**	2 M KOH	3-electrode	176.8 (1)<br>156.0 (10)	88.2%	[19]
**CoO/Co_3O_4**	3 M KOH	3-electrode	451 (1)<br>353 (10)<br>308 (20)	78.3%<br>68.3%	[20]
**Mesoporous Co_3O_4 nanowire**	6 M KOH	3-electrode	1160 (2)<br>884 (10)<br>820 (20)	76.2%<br>70.7%	[21]
**Macro-/ Mesoporous Co_3O_4**	2 M KOH	3-electrode	742.3 (0.5)<br>403.8 (20)	54.4%	[22]
**Carbon Nanofibers/ Co_3O_4**	6 M KOH	3-electrode	586 (1)<br>490 (10)	83.6%	[23]

			196 (50)	33.4%	
**Co_3O_4-CNFs1**	6 M KOH	3-electrode	270 (1)		[24]
			225 (12)	83%	
**Co_3O_4-CNFs2**			325 (1)		
			256 (12)	79%	
**Co_3O_4-CNFs3**			552 (1)		
			403 (12)	73%	
**Mesoporous Co_3O_4 Nanosheet**	30 wt % KOH	2-electrode	905 (1)<br>705.9 (40)	78%	[25]
**UCNG**	2 M KOH	3-electrode	978.1 (1)<br>916.5 (10)	93.7%	This work

Table S4 Comparison of the rate capability of some advanced carbon materials in the literature.

Materials	Electrolyte	Measurement methods	C_s/ F g^{-1} (I_s / A g^{-1})	Rate capability	Ref.
**PCNS-G-4**	6 M KOH	3-electrode	300 (0.5)		[26]
			246 (10)	82%	
	6 M KOH	2-electrode	228 (1)		
			189.2 (40)	83%	
	1 M TEABF4/AN	2-electrode	106 (1)		
			85.9 (40)	81%	
**Shape-Tailorable Graphene**	PVA-Na_2SO_4 gel	2-electrode	From50mV/s to 5000 mV/s	53%	[27]
**Sheet-like porous carbon**	6 M KOH	3-electrode	N/A (~0.5)		[28]
			N/A (~120)	~75%	
		2-electrode	N/A (1)		
			N/A (20)	~89%	
**B/O co-doped carbon nanofiber films**	1 M H_2SO_4	2-electrode	179.3 Fcm^{-3}(1)		[29]
			158.9 Fcm^{-3} (10)	88.6%	
			140.7Fcm^{-3} (100)	78.5%	
**GMCS–NH_3**	6 M KOH	2-electrode	29.6 (0.1)<br>25.2 (25)	85%	[30]
**Functionalized highly porous graphitic carbon fibers**	1 M H_2SO_4	3-electrode	175 (1)		[31]
			107 (60)	61%	
	1 M H_2SO_4	2-electrode	96 (1)		
			48 (120)	50%	
**Single-walled carbon nanotube aerogels**	Ionic liquid	2-electrode	~50 (1)<br>~25 (60)	~50%	[32]
**N-doped porous carbon buildings**	6 M KOH	3-electrode	347 (1)<br>278 (50)	80%	[33]
**UCNG**	2 M KOH	3-electrode	978.1 (1)<br>916.5 (10)	93.7%	This work

Part II: Computation details

Spin-polarized DFT calculations are performed using the Vienna Ab-initio Simulation Package (VASP)[34, 35] with projector augmented wave (PAW) pseudopotentials[36, 37] and the Perdew–Burke–Ernzerhof (PBE) exchange-correlation functional[38]. The electron correlation was remedied by using the LDA+U approach[39], with U=3 eV for Co d-electrons, which has been shown to reproduce well the experimental structural parameters, heat of formation, and the band gap[40]. The atomic structures and the coordinates (in VASP CONTCAR format) used to model the 001 surface are shown in Figure S22. We used 400 eV for the plane-wave cutoff, and fully relaxed the systems until the final force on each atom is less than 0.01 eV/Å. 5x5x1 k-points with Monkhorst-Pack sampling[41] are used to relax the systems, and 21x21x1 k-points are used to calculate the DOS.

```
Perfect 001 surface
  1.00000000000000
    8.1500000000000004   0.0000000000000000   0.0000000000000000
    0.0000000000000000   8.1500000000000004   0.0000000000000000
    0.0000000000000000   0.0000000000000000  20.0000000000000000
  Co   O
   30   40
Selective dynamics
Direct
  0.0000977465099083  0.5002195250814268  0.4046075283703487   T   T   T
  0.9887789289470135  0.0117258177517883  0.2038345628438449   T   T   T
  0.5023683232349327  0.4970064977758497  0.1948311284579987   T   T   T
  0.5981191429062633  0.4112613492843167  0.5834208901865452   T   T   T
  0.4994081384111979  0.0002157725558973  0.4046575308906597   T   T   T
  0.7499952269322563  0.7498403726926313  0.3085249846230553   T   T   T
  0.7615181750050724  0.2384331599526917  0.5093137663493010   T   T   T
  0.1524743769208570  0.8390744881178236  0.1296150300278143   T   T   T
  0.2478274734410491  0.7535835383290532  0.5181560487454746   T   T   T
  0.2507875785055091  0.2497860902984570  0.3084268406271633   T   T   T
  0.6213815398808222  0.8875027057333469  0.5598089327490712   T   T   T
  0.8745798731743477  0.6290925847810129  0.5579467360663486   T   T   T
  0.1166906777901602  0.3806795055406624  0.5597277673411512   T   T   T
  0.3766462922002702  0.1268741391400638  0.5593929459992850   T   T   T
  0.6337575473441674  0.8696813852505301  0.1533943933350344   T   T   T
  0.8758399838625337  0.6211595507435916  0.1550269714047445   T   T   T
  0.1289379588617692  0.3627960270605897  0.1533362505992955   T   T   T
  0.3738595014516335  0.1234613856084437  0.1536459109143777   T   T   T
  0.6230030374947546  0.6263949303774581  0.4577607184063979   T   T   T
  0.3750774566133330  0.8733507950506763  0.2547088164029816   T   T   T
  0.6242669527868969  0.3754988831195973  0.3561967674292177   T   T   T
  0.6230081195592660  0.1254905974306837  0.2545645868712256   T   T   T
  0.3750924474074111  0.3767398614773612  0.4585086948446957   T   T   T
  0.8750472729849363  0.1250754521251665  0.3566314018900627   T   T   T
  0.1256555068917962  0.8747060436295442  0.3570548892220629   T   T   T
  0.1270606226596698  0.6238358442588350  0.2554732034516576   T   T   T
  0.8753111312998456  0.8733023951612537  0.4585644763965533   T   T   T
  0.3750267250762676  0.6250283424585774  0.3566723690771951   T   T   T
  0.1272185735927351  0.1247335457515035  0.4586278600075602   T   T   T
  0.8748331935504154  0.3768615338756121  0.2546209305953511   T   T   T
  0.1397991364182474  0.3628815775396390  0.4601332376677973   T   T   T
  0.1141238925238781  0.6114342803956134  0.1573588355006450   T   T   T
  0.1026444026643247  0.6090187979588109  0.5684331610285724   T   T   T
  0.8926823328570848  0.6110836320945268  0.2556751448873911   T   T   T
```

```
  0.8948559152574731  0.3884183222383513  0.1575368757208295   T   T   T
  0.8571049428020387  0.3973369055710094  0.5741834148027962   T   T   T
  0.8573980716919536  0.6390882794462058  0.4573653594279250   T   T   T
  0.8933218792065958  0.8529083280044105  0.1388333121599388   T   T   T
  0.8555156873096763  0.8617965789459774  0.5555561266762439   T   T   T
  0.6103961313713668  0.8872656087349569  0.2530049831840770   T   T   T
  0.6477258352825999  0.6414137226815981  0.1444685077290089   T   T   T
  0.6363193147738144  0.6388642117142851  0.5558599528350570   T   T   T
  0.1372349637163595  0.8629894824570741  0.2593739790908955   T   T   T
  0.1101951181002576  0.1094205137968203  0.3626145386845394   T   T   T
  0.8918306324928551  0.1086011174807524  0.4525311870753228   T   T   T
  0.8908355416944431  0.8899624102709396  0.3627299398110750   T   T   T
  0.8583953238096811  0.1415568211053824  0.2606488222266776   T   T   T
  0.8591850529948175  0.3602385738024054  0.3504668227260765   T   T   T
  0.6128889963158315  0.3872031139388454  0.4539016050611124   T   T   T
  0.6398749183752273  0.1407585591170957  0.3505796424492473   T   T   T
  0.6359871152233225  0.3638865829091884  0.2586276154913989   T   T   T
  0.6089166048219141  0.6104141678874910  0.3628693758407522   T   T   T
  0.3859314569471692  0.6142017468535883  0.4547462087255880   T   T   T
  0.3891728999047288  0.3908935283258899  0.3634602685557553   T   T   T
  0.3641887272138362  0.6359263720474715  0.2585215363169908   T   T   T
  0.3608457773747560  0.8591812541868862  0.3497612878927114   T   T   T
  0.1140441756409700  0.8864169336822698  0.4545317813514913   T   T   T
  0.1410874133068560  0.6397574729048827  0.3503904581202377   T   T   T
  0.6386533727298200  0.8597181003749341  0.4589987782513347   T   T   T
  0.6070283914004619  0.1024031536638716  0.1589206555295704   T   T   T
  0.6080133264732197  0.1331791394152546  0.5731383768562068   T   T   T
  0.3893605577988524  0.1078301427287798  0.2552022449242699   T   T   T
  0.3787794829725613  0.8866067073443631  0.1553242524687874   T   T   T
  0.3924856664650918  0.8984226349809035  0.5684698123272440   T   T   T
  0.3608673318647249  0.1422898337986709  0.4578675453347003   T   T   T
  0.3578101400149620  0.3519778251098771  0.1444823496588654   T   T   T
  0.3717267103044364  0.3636096174237409  0.5577952908745232   T   T   T
  0.1115151786488795  0.3904870471474169  0.2541644638767835   T   T   T
  0.1424828045837288  0.1169904956609926  0.1399446725787641   T   T   T
  0.1434610795749691  0.1478966171327798  0.5542246421523060   T   T   T
```

V_O

```
   1.00000000000000
     8.1500000000000004    0.0000000000000000    0.0000000000000000
     0.0000000000000000    8.1500000000000004    0.0000000000000000
     0.0000000000000000    0.0000000000000000   20.0000000000000000
   Co   O
    30    38
Selective dynamics
Direct
  0.9961315508177506  0.5022073966592941  0.4061240360688458   T   T   T
  0.9892873468152246  0.9984732850918974  0.1954250307669341   T   T   T
  0.4984460250984455  0.4905060731025301  0.1939799879732647   T   T   T
  0.5279761256679194  0.5248535944875528  0.5787047469174240   T   T   T
  0.5006565218426289  0.9941360116212437  0.4085527026692972   T   T   T
  0.7448105907904932  0.7511038088600444  0.3046935352599363   T   T   T
  0.7479574955687909  0.2398637374642831  0.5195847452728373   T   T   T
  0.2712216941553294  0.7819255044989930  0.1341000668653010   T   T   T
  0.2393070114978784  0.7489281450925560  0.5170256198995489   T   T   T
  0.2515407136290335  0.2456874224497909  0.3068867447992716   T   T   T
  0.5979785516528509  0.8764152325564893  0.5642119829780725   T   T   T
  0.8833363680339446  0.6040775204115860  0.5641526858269259   T   T   T
  0.1227819986432195  0.3740492162637636  0.5575200156970226   T   T   T
  0.3713601709485985  0.1232770962040703  0.5581998620281539   T   T   T
  0.6317992763160802  0.8529706894229463  0.1480681073517118   T   T   T
  0.8738016345380970  0.6229201414129832  0.1552332153461649   T   T   T
  0.1227007684187882  0.3725527576346650  0.1550621415007285   T   T   T
  0.3501288155164559  0.1278717752961711  0.1499728427386344   T   T   T
  0.6240089739845160  0.6226212495766106  0.4592224094111330   T   T   T
  0.3740686756916247  0.8725069873125975  0.2541410384956251   T   T   T
  0.6247123779315586  0.3744122219471251  0.3568422219004717   T   T   T
  0.6223407976287376  0.1234430033169005  0.2582371118187936   T   T   T
  0.3758207278870174  0.3744475014775261  0.4567269811222090   T   T   T
```

0.8733712217053053	0.1249128350470770	0.3565175143808119	T	T	T
0.1246179177385756	0.8747851023451219	0.3563540891603765	T	T	T
0.1265186211844593	0.6244174131967881	0.2564589393368166	T	T	T
0.8726535563782392	0.8731105990417802	0.4547872197396359	T	T	T
0.3759904374688858	0.6241727261078225	0.3567645767693932	T	T	T
0.1234097406623818	0.1258938494401747	0.4581831889575554	T	T	T
0.8745688937546348	0.3753437019482533	0.2549484757286535	T	T	T
0.1425706080427389	0.3619093950242913	0.4572144075705182	T	T	T
0.1179122958088072	0.6165628830813645	0.1582693869647187	T	T	T
0.1170915058711444	0.5984528990084854	0.5722859786635937	T	T	T
0.8935095529698529	0.6110182467736180	0.2558173959300234	T	T	T
0.8923415322614687	0.3931796101274898	0.1586073004558131	T	T	T
0.8895566421116285	0.3647792945144630	0.5654198959998240	T	T	T
0.8599084185115586	0.6432297303348804	0.4646740157701856	T	T	T
0.8662236320518630	0.8476307508183822	0.1407107145281827	T	T	T
0.6094505063043414	0.8936934618322709	0.2478040221801550	T	T	T
0.6413047826043865	0.6136049077603758	0.1478288225761659	T	T	T
0.6796340382728161	0.6762106497194296	0.6041617892984377	T	T	T
0.1366691640520088	0.8625835484923883	0.2589244434893416	T	T	T
0.1079312479337133	0.1092298037217390	0.3623224824255260	T	T	T
0.8826700494888158	0.1110959573528518	0.4539174067590750	T	T	T
0.8874591546338948	0.8895022260665471	0.3606756488284475	T	T	T
0.8583687688609416	0.1368199214600665	0.2592820581976127	T	T	T
0.8585071049562600	0.3593467828797046	0.3507588364240846	T	T	T
0.6164799010233182	0.3830595524794518	0.4542181658634945	T	T	T
0.6381013760749568	0.1379205492707314	0.3522861271798021	T	T	T
0.6330118558702509	0.3608242328051574	0.2592719820521197	T	T	T
0.6108916256615373	0.6094469340264013	0.3608480652418180	T	T	T
0.3864613449472998	0.6124423307838356	0.4541781815515122	T	T	T
0.3906234247145832	0.3888670137156183	0.3621219669854980	T	T	T
0.3666030124215425	0.6330253175206124	0.2591168475416055	T	T	T
0.3603642785241021	0.8591238281063056	0.3525613703922588	T	T	T
0.1076593871182041	0.8870810601511252	0.4538410638632300	T	T	T
0.1403891958080408	0.6400265673124252	0.3512720821924944	T	T	T
0.6423679779863178	0.8569596085612261	0.4650497214280023	T	T	T
0.6055762241507097	0.1148994204554796	0.5672502622210374	T	T	T
0.3919942081812948	0.1074672418683917	0.2488640877582355	T	T	T
0.4288283718175734	0.9269582735218691	0.1087101295356803	T	T	T
0.3632453641481632	0.8994337665072010	0.5701628545310697	T	T	T
0.3579996263723046	0.1407165770324568	0.4569671407276275	T	T	T
0.3564682425793464	0.3658558842086350	0.1457514787896130	T	T	T
0.3676372602488271	0.3659480418920253	0.5550824922113620	T	T	T
0.1093417797265559	0.3903156753985968	0.2561853257854665	T	T	T
0.1154217571357563	0.1480851781892767	0.1429140739794477	T	T	T
0.1407336757181241	0.1445486092245503	0.5544251373253530	T	T	T

References

[1] Z.-S. Wu, W. Ren, L. Wen, L. Gao, J. Zhao, Z. Chen, G. Zhou, F. Li, H.-M. Cheng, *ACS Nano* **2010**, *4*, 3187.
[2] S. Yang, X. Song, P. Zhang, J. Sun, L. Gao, *Small* **2014**, *10*, 2270.
[3] X. Li, G. Zhang, X. Bai, X. Sun, X. Wang, E. Wang, H. Dai, **2008**, *3*, 538.
[4] G. Zhou, D.-W. Wang, L.-C. Yin, N. Li, F. Li, H.-M. Cheng, *ACS Nano* **2012**, *6*, 3214.
[5] W.-H. Ryu, T.-H. Yoon, S. H. Song, S. Jeon, Y.-J. Park, I.-D. Kim, *Nano Lett.* **2013**, *13*, 4190.
[6] S. Yang, Y. Lin, X. Song, P. Zhang, L. Gao, *ACS Appl. Mater. Interfaces* **2015**, *7*, 17884.
[7] C. Yuan, L. Yang, L. Hou, L. Shen, X. Zhang, X. W. D. Lou, *Energy Environ. Sci.* **2012**, *5*, 7883.
[8] L. Xie, F. Su, L. Xie, X. Li, Z. Liu, Q. Kong, X. Guo, Y. Zhang, L. Wan, K. Li, C. Lv, C. Chen, *ChemSusChem* **2015**, *8*, 2917.
[9] N. Wang, Q. Liu, D. Kang, J. Gu, W. Zhang, D. Zhang, *ACS Appl. Mater. Interfaces* **2016**, *8*, 16035.
[10] C. Xiang, M. Li, M. Zhi, A. Manivannan, N. Wu, *J. Power Sources* **2013**, *226*, 65.
[11] Y. Xiao, S. Liu, F. Li, A. Zhang, J. Zhao, S. Fang, D. Jia, *Adv. Funct. Mater.* **2012**, *22*, 4052.
[12] Y. Wang, T. Zhou, K. Jiang, P. Da, Z. Peng, J. Tang, B. Kong, W. B. Cai, Z. Yang, G. Zheng, *Adv. Energy Mater.* **2014**, *4*.
[13] J. Li, Z. Liu, L. Li, C. Zhu, D. Hu, *J. Electrochem. Soc.* **2016**, *163*, A417.
[14] F. Su, X. Lv, M. Miao, *Small* **2015**, *11*, 854.
[15] C. Feng, J. Zhang, Y. He, C. Zhong, W. Hu, L. Liu, Y. Deng, *ACS Nano* **2015**, *9*, 1730.
[16] Q. Liao, N. Li, S. Jin, G. Yang, C. Wang, *ACS Nano* **2015**, *9*, 5310.
[17] D. Yan, H. Zhang, L. Chen, G. Zhu, S. Li, H. Xu, A. Yu, *ACS Appl. Mater. Interfaces* **2014**, *6*, 15632.
[18] Q. Guan, J. Cheng, B. Wang, W. Ni, G. Gu, X. Li, L. Huang, G. Yang, F. Nie, *ACS Appl. Mater. Interfaces* **2014**, *6*, 7626.
[19] Y. Wang, Z. Zhong, Y. Chen, C. T. Ng, J. Lin, *Nano Res.* **2011**, *4*, 695.
[20] M. Pang, G. Long, S. Jiang, Y. Ji, W. Han, B. Wang, X. Liu, Y. Xi, D. Wang, F. Xu, *Chem. Eng. J.* **2015**, *280*, 377.
[21] F. Zhang, C. Yuan, X. Lu, L. Zhang, Q. Che, X. Zhang, *J. Power Sources* **2012**, *203*, 250.
[22] X. Wang, A. Sumboja, E. Khoo, C. Yan, P. S. Lee, *J. Phys. Chem. C* **2012**, *116*, 4930.
[23] S. Abouali, M. Akbari Garakani, B. Zhang, Z.-L. Xu, E. Kamali Heidari, J.-q. Huang, J. Huang, J.-K. Kim, *ACS Appl. Mater. Interfaces* **2015**, *7*, 13503.
[24] F. Zhang, C. Yuan, J. Zhu, J. Wang, X. Zhang, X. W. D. Lou, *Adv. Funct. Mater.* **2013**, *23*, 3909.
[25] R. Rakhi, W. Chen, M. N. Hedhili, D. Cha, H. N. Alshareef, *ACS Appl. Mater. Interfaces* **2014**, *6*, 4196.
[26] X. Fan, C. Yu, J. Yang, Z. Ling, C. Hu, M. Zhang, J. Qiu, *Adv. Energy Mater.* **2015**, *5*.
[27] B. Xie, C. Yang, Z. Zhang, P. Zou, Z. Lin, G. Shi, Q. Yang, F. Kang, C.-P. Wong, *ACS Nano* **2015**, *9*, 5636.
[28] Z. Li, W. Lv, C. Zhang, B. Li, F. Kang, Q.-H. Yang, *Carbon* **2015**, *92*, 11.
[29] Z.-Y. Yu, L.-F. Chen, L.-T. Song, Y.-W. Zhu, H.-X. Ji, S.-H. Yu, *Nano Energy* **2015**, *15*, 235.
[30] Z. Lei, N. Christov, X. Zhao, *Energy Environ. Sci.* **2011**, *4*, 1866.
[31] H. Wang, H. Yi, C. Zhu, X. Wang, H. Jin Fan, *Nano Energy* **2015**, *13*, 658.

[32] K. L. Van Aken, C. R. Pérez, Y. Oh, M. Beidaghi, Y. Joo Jeong, M. F. Islam, Y. Gogotsi, *Nano Energy* **2015**, *15*, 662.
[33] L. Jiang, L. Sheng, X. Chen, T. Wei, Z. Fan, *J. Mater. Chem. A* **2016**, *4*, 11388.
[34] G. Kresse, J. Hafner, *Phys. Rev. B* **1993**, *47*, 558.
[35] G. Kresse, J. Furthmüller, *Phys. Rev. B* **1996**, *54*, 11169.
[36] G. Kresse, D. Joubert, *Phys. Rev. B* **1999**, *59*, 1758.
[37] P. E. Blöchl, *Phys. Rev. B* **1994**, *50*, 17953.
[38] J. P. Perdew, K. Burke, M. Ernzerhof, *Phys. Rev. Lett.* **1996**, *77*, 3865.
[39] S. L. Dudarev, G. A. Botton, S. Y. Savrasov, C. J. Humphreys, A. P. Sutton, *Phys. Rev. B* **1998**, *57*, 1505.
[40] G. A. Kaptagay, T. M. Inerbaev, Y. A. Mastrikov, E. A. Kotomin, A. T. Akilbekov, *Solid State Ionics* **2015**, *277*, 77.
[41] H. J. Monkhorst, J. D. Pack, *Phys. Rev. B* **1976**, *13*, 5188.
